# Metal‐Dependent and Selective Crystallization of CAU‐10 and MIL‐53 Frameworks through Linker Nitration

**DOI:** 10.1002/chem.202100373

**Published:** 2021-03-10

**Authors:** Timo Rabe, Erik Svensson Grape, Tobias A. Engesser, A. Ken Inge, Jonas Ströh, Gitta Kohlmeyer‐Yilmaz, Mohammad Wahiduzzaman, Guillaume Maurin, Frank D. Sönnichsen, Norbert Stock

**Affiliations:** ^1^ Department of Inorganic Chemistry Christian-Albrechts-Universität zu Kiel 24118 Kiel Germany; ^2^ Department of Materials and Environmental Chemistry Stockholm University 10691 Stockholm Sweden; ^3^ Otto Diels Institute for Organic Chemistry Christian-Albrechts-Universität zu Kiel 24118 Kiel Germany; ^4^ ICGM, Univ. Montpellier CNRS ENSCM Université Montpellier 34095 Montpellier France

**Keywords:** aluminium, framework flexibility, gallium, molecular simulations, water adsorption

## Abstract

The reaction of the V‐shaped linker molecule 5‐hydroxyisophthalic acid (H_2_L^0^), with Al or Ga nitrate under almost identical reaction conditions leads to the nitration of the linker and subsequent formation of metal–organic frameworks (MOFs) with CAU‐10 or MIL‐53 type structure of composition [Al(OH)(L)], denoted as Al‐CAU‐10‐L^0, 2, 4, 6^ or [Ga(OH)(L)], denoted as Ga‐MIL‐53‐L^2^. The Al‐MOF contains the original linker L^0^ as well as three different nitration products (L^2^, L^4^ and L^4/6^), whereas the Ga‐MOF mainly incorporates the linker L^2^. The compositions were deduced by ^1^H NMR spectroscopy and confirmed by Rietveld refinement. In situ and ex situ studies were carried out to follow the nitration and crystallization, as well as the composition of the MOFs. The crystal structures were refined against powder X‐ray diffraction (PXRD) data. As anticipated, the use of the V‐shaped linker results in the formation of the CAU‐10 type structure in the Al‐MOF. Unexpectedly, the Ga‐MOF crystallizes in a MIL‐53 type structure, which is usually observed with linear or slightly bent linker molecules. To study the structure directing effect of the in situ nitrated linker, pure 2‐nitrobenzene‐1,3‐dicarboxylic acid (*m*‐H_2_BDC‐NO_2_) was employed which exclusively led to the formation of [Ga(OH)(C_8_H_3_NO_6_)] (Ga‐MIL‐53‐*m*‐BDC‐NO_2_), which is isoreticular to Ga‐MIL‐53‐L^2^. Density Functional Theory (DFT) calculations confirmed the higher stability of Ga‐MIL‐53‐L^2^ compared to Ga‐CAU‐10‐L^2^ and grand canonical Monte Carlo simulations (GCMC) are in agreement with the observed water adsorption isotherms of Ga‐MIL‐53‐L^2^.

## Graphical abstract

**In the frame**: The nitration of the V‐shaped linker 5‐hydroxyisophthalic acid by Al(NO_3_)_3_ and Ga(NO_3_)_3_ prior to crystallization of the MOF leads selectively to Al‐CAU‐10 and Ga‐MIL‐53 type compounds. In situ IR spectroscopy and light scattering experiments in combination with ^1^H‐NMR spectroscopy were used to study the product formation. DFT and GCMC calculations were employed to understand the differences in framework formation and water adsorption properties.

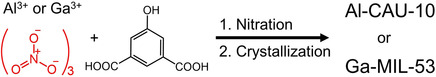

## Introduction

Over the past years, metal–organic frameworks (MOFs) have been one of the most intensively studied classes of materials in inorganic chemistry.^[1]^ The typically crystalline solids, are constructed from inorganic (metal ions or metal‐oxygen clusters) and organic building units (ligands or linkers). The modularity leads to materials which can assemble into various framework topologies.^[2]^ Inorganic and organic components are often selectively chosen in order to create materials with defined pore networks and desired properties.^[3]^ Through this approach MOFs have become a well‐studied class of porous materials for applications in fields such as heat transformation,^[4]^ drug delivery,^[5]^ gas storage^[6]^ and catalysis.^[7]^

Al‐MOFs like CAU‐10^[8]^ [Al(OH)(*m*‐BDC)] (with *m*‐H_2_BDC=isophthalic acid), among others, have been studied intensively in the last decade due to the availability of aluminium, their high chemical and thermal stability and outstanding sorption properties,^[9]^ but compounds with the heavier homologue gallium have rarely been reported.^[10]^ Synthesis conditions of Al‐ and Ga‐MOFs are largely similar and the probability to acquire isoreticular frameworks is very high, for example in the prominent MIL‐53 type structure [M(OH)(*p*‐BDC)] (M=Al^3+^, Ga^3+^)^[11]^ with *p*‐H_2_BDC=terephthalic acid or [M(OH)(fum)] (M=Al^3+^, Ga^3+^, In^3+^)^[12]^ with H_2_fum=fumaric acid.^[10]^ Hence, there are only a few examples of unique structures reported for Ga‐MOFs, although they are known to exhibit interesting properties, therefore showing the need for further investigations.[^10^, ^13^]

The in situ formation of ligands during MOF synthesis under solvothermal reaction conditions is a powerful tool in the assembly of unique frameworks and can open up new strategic routes for MOFs that may be inaccessible via direct preparation.^[14]^ Classical reactions include for example hydrolysis,^[15]^ hydroxylation,^[16]^ alkylation,^[17]^ decarboxylation,^[18]^ acylation^[19]^ or nitration. Decarboxylation and nitration of 5‐hydroxyisophthalic acid (H_2_L^0^) during the synthesis of homo‐ and heterometallic coordination polymers have been reported previously.^[20]^ Nitration of H_2_L^0^ with nitrating acid leads to mixtures of mono‐nitrated compounds in the 2‐ and 4‐position at lower temperatures. Higher temperatures favour di‐ or trinitrated product mixtures. In addition decarboxylation of H_2_L^0^ can occur.[^20b^, ^21^]

Here we report our latest results on the systematic investigation of Al‐ and Ga‐MOFs employing the linker 5‐hydroxyisophthalic acid under solvothermal reaction conditions. In situ nitration and assembly into CAU‐10 and MIL‐53 type structures for Al and Ga, respectively, is demonstrated, showing the direct influence of the metal source on the framework formation and functionalization.

## Results and Discussion

**Figure 1:**
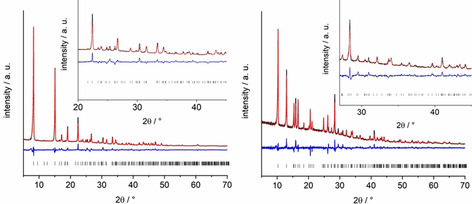
Final Rietveld plots of Al‐CAU‐10‐L^0, 2, 4, 6^ (left) and Ga‐MIL‐53‐L^2^_np (right). In black the experimental pattern, in red the calculated pattern, the difference in blue and the allowed reflections as black lines.

**Figure 2:**
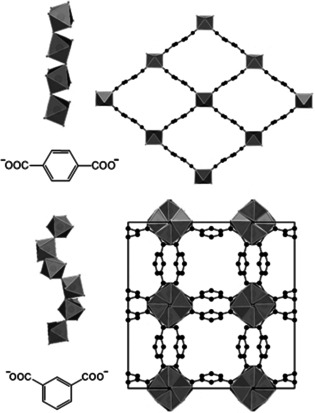
IBU and framework structure of [Al(OH)(*p*‐BDC)] and [Al(OH)(*m*‐BDC)] crystallizing in the MIL‐53 (top) and CAU‐10 (bottom) structures, respectively.[^8^, ^11a^] Al(OH)_2_O_4_‐polyhedra in grey, oxygen in red and carbon as well as the unit cell edges in black.

The reaction of 5‐hydroxy‐isophthalic acid (H_2_L^0^) with Al(NO_3_)_3_ or Ga(NO_3_)_3_ in a mixture of water/acetic acid resulted in the formation of two different MOFs crystallizing in the well‐known CAU‐10 and MIL‐53 type structures.^[22]^ The reaction products were obtained as microcrystalline powders and PXRD data had to be used for the structure refinement (Figure 1). Crystal structure data are summarized in Table 1. In the following section, the framework structures of the parent Al‐MOFs are briefly described (Figure 2). Both frameworks are composed of 1D inorganic building units (IBUs) that are connected to four other chains by the dicarboxylate ions. While in MIL‐53 chains of *trans* corner sharing polyhedra are found, *cis* corner sharing of the polyhedra leads to the helical IBU in CAU‐10.

**Table 1:** Crystallographic data for Al‐CAU‐10‐L^0, 2, 4, 6^, Ga‐MIL‐53‐L^2^_lp1, Ga‐MIL‐53‐L^2^_lp2, Ga‐MIL‐53‐L^2^_np as well as Ga‐MIL‐53‐*m*‐BDC‐NO_2_.

	Al‐CAU‐10‐L^0, 2, 4, 6^	Ga‐MIL‐53‐L^2^_lp1	Ga‐MIL‐53‐L^2^_lp2	Ga‐MIL‐53‐L^2^_np	Ga‐MIL‐53‐*m‐*BDC‐NO_2_
refinement	Rietveld	Le Bail	Rietveld	Rietveld	Rietveld
crystal system	tetragonal	orthorhombic	orthorhombic	orthorhombic	orthorhombic
space group	*I*4_1_/*amd* *(No. 141)*	*Pna*2_1_ *(No. 33)*	*Pnma* *(No. 62)*	*Pnma* *(No. 62)*	*Pnma* *(No. 62)*
*a* [Å]	21.5030(9)	14.771(1)	14.9265(10)	13.6369(6)	14.6254(5)
*b* [Å]	21.5030(9)	6.7458(6)	6.7749(3)	6.7567(3)	6.7673(2)
*c* [Å]	10.2952(6)	12.487(1)	11.1121(8)	11.1511(6)	10.5282(5)
*V* [Å^3^]	4759.2(5)	1244.3(2)	1123.8(1)	1027.47(5)	1042.03(7)
*R_wp_* [%]	5.69	2.55	8.73	4.79	7.20
*R_Bragg_* [%]	3.30	–	4.42	3.46	5.98
GoF	4.29	1.85	4.19	2.78	6.95

The linker shape is typically the major factor determining the connectivity of the AlO_6_ polyhedra. Linear linker molecules usually result in the formation of MIL‐53 type structures, while V‐shaped linkers form the CAU‐10 type structure. Surprisingly in this study, the two framework structures are obtained from V‐shaped linker molecules. To the best of our knowledge, until now no MIL‐53 type structure containing a V‐shaped linker with an angle of 120° between the carboxylate groups of the linker has been reported. Only 2,5‐thiophendicarboxylic acid (H_2_TDC, 148°) and (+)‐camphoric acid (H_2_CAM, 139°) have been shown to yield MIL‐53 type frameworks ([M(OH)(TDC)] M=Al^3+^, Ga^3+^, In^3+^ and [Ga(OH)(CAM)]).^[23]^ The two title compounds were obtained under almost identical solvothermal synthesis conditions depending on the metal ion employed.

The use of Al(NO_3_)_3_ and Ga(NO_3_)_3_ as reactants resulted in compounds of composition [Al(OH)(C_8_H_2.08_O_5_(NO_2_)_0.92_)] and [Ga(OH)(C_8_H_2_O_3_(NO_2_)] crystallizing in the CAU‐10 and MIL‐53 type structure, respectively. Since nitrated linkers are found in the final reaction products, in situ nitration of the linker H_2_L_0_ must take place. Therefore the synthesis of the two MOFs was studied in more detail by in situ IR spectroscopy combined with light scattering (Figure S1–S4 in Supporting Information).

**Figure 3:**
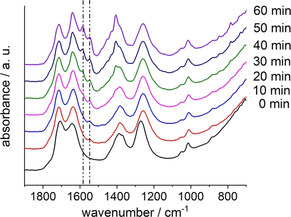
In situ IR spectra of the reaction mixture for the synthesis of Al‐CAU‐10‐L^0, 2, 4, 6^ after 0, 10, 20, 30, 40, 50 and 60 min at 120 °C. The vibrational bands which can be assigned to the antisymmetric ‐NO_2_ stretching and the antisymmetric ‐CO_2_^−^ stretching vibrational bands are marked by dashed lines at 1550 and 1580 cm^−1^, respectively.

The data confirms the nitration of the ligand H_2_L^0^ prior to the framework formation (Figure 3). The ligand is completely dissolved within 1 min and 20 seconds at a reaction temperature of 120 °C and a clear, transparent solution was formed, which started to turn slightly yellow after 2 min (Figure S2). After 5 min the solution turned intensively yellow and a band at 1550 cm^−1^, which can be assigned to the anti‐symmetric stretching vibration of the aromatic nitro group, is clearly observed (Figure 3, Figures S3 and S4). The intensity of this band increases with reaction time and after 30 min the vibrational band of the anti‐symmetric stretching vibration of the carboxylate group becomes visible. This is in agreement with the Faraday–Tyndall effect first observed after 30 min, triggered by colloidal particles in solution, showing the start of product formation (Figure S2).^[24]^

**Figure 4:**
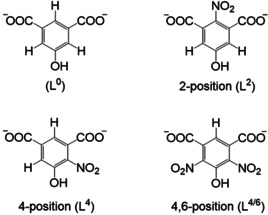
5‐Hydroxyisophthalic acid (H_2_L^0^, top left) and nitration products of 5‐hydroxyisophthalic acid (H_2_L^0^) observed in our investigation. The position of the nitro group indicated in the superscript L^*x*^, *x=*2, 4, 6.

^1^H NMR spectroscopy was used to quantitatively determine the reaction products formed during the nitration reaction (Figure S5) and the nitrated ligands incorporated into the final framework structures. Electrophilic aromatic substitution at different positions of the aromatic ring resulting in the formation of the nitrated linker molecules as shown in Figure 4 was revealed.

The linker distribution in the two MOFs was also determined by NMR spectroscopy. Ex situ analyses of reactions quenched after 1, 2 and 24 h were carried out. The reaction products were dissolved in a mixture of NaOD/D_2_O and ^1^H NMR as well as ^1^H ^13^C HMBC NMR spectra of the Ga‐ and Al‐MOFs were recorded (Figure S6 and S7). The detailed evaluation demonstrated the incorporation of the different dicarboxylate molecules L^0^, L^2^, L^4^ and L^4/6^ into the framework of Al‐CAU‐10 while mainly the linker L^2^ was observed in Ga‐MIL‐53. Hence the compounds are denoted as Al‐CAU‐10‐L^0, 2, 4, 6^ and Ga‐MIL‐53‐L^2^, respectively. Whereas the relative intensities of the differently nitrated ligands did not change with reaction time in Al‐CAU‐10‐L^0, 2, 4, 6^ (Figure S8), an increase in the amount of incorporated linker L^2^ from 47 % after 1 h to 92 % after 24 h is observed for Ga‐MIL‐53‐L^2^ (Figure S9 and Table S1). This is most likely related to the higher lability and faster ligand exchange rate of Ga^3+^ ions as well as the steric hindrance in the 2‐position when forming the MIL‐53 type framework.

The Rietveld refinements confirm the incorporation of the different nitrated linker molecules. For Al‐CAU‐10‐L^0, 2, 4, 6^ the Rietveld refinements resulted in a statistical incorporation of different linkers (L^0^ (15 %), L^2^ (7 %), L^4/6^ (7 %) and L^4^ (35.5 %)) and an average total occupancy of 0.92 nitro groups per linker was determined. Accordingly the results of the Rietveld refinement of Ga‐MIL‐53‐L^2^ showed the predominant incorporation of L^2^ (92 %) and the absence of the unfunctionalized linker L^0^.

As mentioned above, despite the almost identical synthetic conditions, the use of Al(NO_3_)_3_ or Ga(NO_3_)_3_ as reactants leads to MOFs with two distinct framework topologies. In order to better understand this peculiarity, the DFT electronic energies of the ground state geometries for all linker variants of the Ga‐MOFs were analysed (details are given in the Supporting Information, section 3). The Ga‐MIL‐53_lp1 geometry was optimized with fixed cell parameters as obtained from the indexing of the PXRD data. In addition, two hypothetical Ga‐MOFs were constructed based on the Al‐CAU‐10‐L^0, 2, 4, 6^ structure. These, Ga‐CAU‐10 structures (Ga‐CAU‐10‐L^2^ and Ga‐CAU‐10‐L^4^) were then fully relaxed, that is, both the cell parameters and atomic positions were allowed to relax simultaneously during the geometry optimization. The MIL‐53 framework is energetically more stable than Ga‐CAU‐10 for both mono functionalized L^2^ and L^4^ linkers with ≈0.50 kcal mol^−1^ atom^−1^ and ≈0.32 kcal mol^−1^ atom^−1^, respectively, consistent with the experimental findings. The Ga‐MIL‐53‐L^4^ structure is energetically more stable than its L^2^ isomer by ≈0.16 kcal mol^−1^ atom^−1^. Geometrically, this energy gain is attributed to additional OH⋅⋅⋅NO_2_ interactions, depending on the orientation of ‐NO_2_ of the L^4^ linkers. The latter results of the DFT calculations stand in contrast to the experimental findings where mainly the L^2^ isomer and only small amounts of the L^4^ isomer are observed in the final reaction product. This suggests that the linker incorporation during the crystal growth of a MOF is most probably a combined thermodynamic and kinetically driven process.

**Figure 5:**
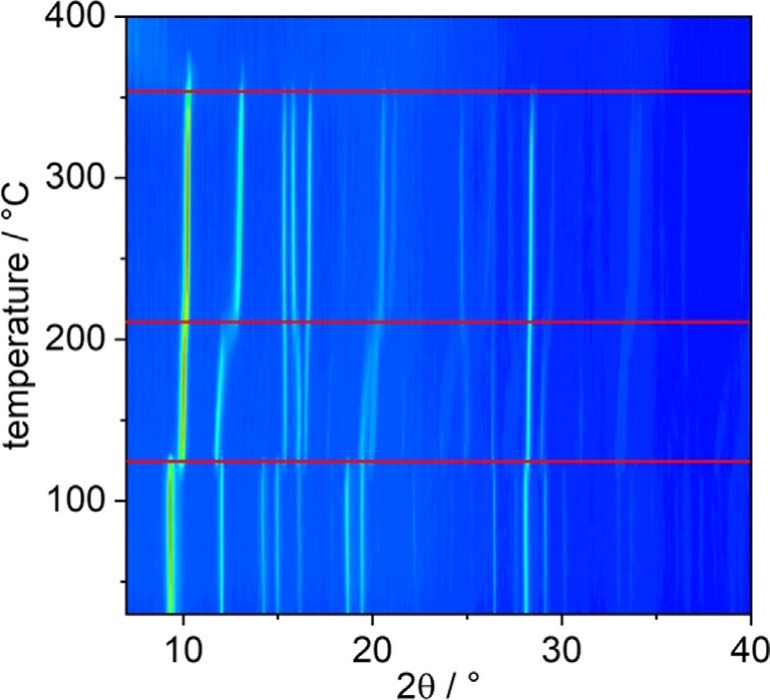
Results of the TD PXRD measurement of Ga‐MIL‐53‐L^2^ with red lines indicating the phase transformation from Ga‐MIL‐53‐L^2^_lp1 to Ga‐MIL‐53‐L^2^_lp2 (at 125 °C) to Ga‐MIL‐53‐L^2^_np (at 205 °C) and the decomposition of the framework (at 350 °C).

Al and Ga‐MOFs crystallizing in the MIL‐53 and CAU‐10 type structures are known to exhibit framework flexibility. While only subtle changes related to linker rotations are observed in CAU‐10, unit cell volume changes of up to 50 % have been reported for Al‐MIL‐53‐*p*‐BDC.^[11a]^ These structural changes are readily detected by temperature dependent (TD) PXRD measurements, which were recorded for Al‐CAU‐10‐L^0, 2, 4, 6^ and Ga‐MIL‐53‐L^2^ in a temperature range of 30–500 °C using open quartz capillaries (Figure 5 and Figure S10). Surprisingly for Al‐CAU‐10‐L^0, 2, 4, 6^ no structural changes related to the loss of guest molecules is observed and decomposition of the framework takes place around 350 °C (Figure S10). This observation stands in contrast to results of previous studies on CAU‐10 type compounds where the ad‐/desorption of water molecules leads to changes in space group symmetry due to the rotation of the linker molecules.^[8]^ The lack of structural changes in Al‐CAU‐10‐L^0, 2, 4, 6^ is probably due to steric reasons, that is, the presence of ‐NO_2_ groups. The results of the TD PXRD study of Ga‐MIL‐53‐L^2^ are shown in Figure 5 and three distinct structural changes are observed resulting in three different Ga‐MIL‐53‐L^2^ phases, which are denoted as Ga‐MIL‐53‐L^2^_lp1 (T<125 °C), Ga‐MIL‐53‐L^2^_lp2 (125 °C<T<205 °C) and Ga‐MIL‐53‐L^2^_np (205 °C< T<350 °C). Above 350 °C, framework decomposition takes place.

**Figure 6:**
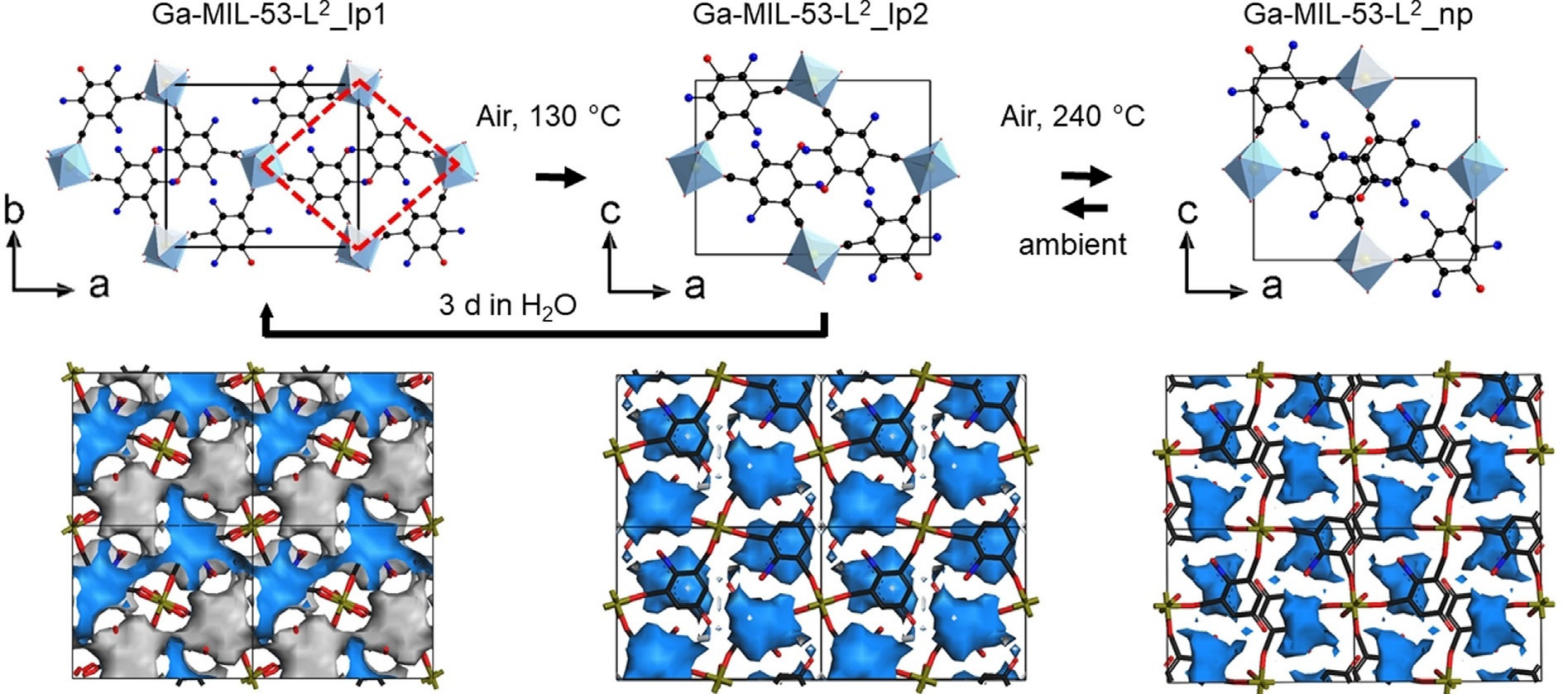
Top: Sections of the crystal structures of the three crystal forms of Ga‐MIL‐53‐L^2^ (oxygen atoms of nitro groups have been omitted for clarity). Arrows indicate the experimental conditions of the phase transformations. Dashed red lines emphasize the interconnection of the IBUs the three Ga compounds. Bottom: Visualization of the pore space of a 2×2×1 supercell, in the crystal structures of Ga‐MIL‐53‐L^2^_lp1, Ga‐MIL‐53‐L^2^_lp2 and Ga‐MIL‐53‐L^2^_np. View along [0 1 0] for Ga‐MIL‐53‐L^2^_lp1 and [0 0 1] for Ga‐MIL‐53‐L^2^_lp2 and Ga‐MIL‐53‐L^2^_np. Blue and grey surfaces mark the inner and outer pore surfaces. Ga(OH)_2_O_4_‐polyhedra in pale blue, oxygen in red, nitrogen in blue and carbon as well as unit cell edges in black.

The MIL‐53 family is well known for its complex structural behaviour upon ad‐ and desorption of guest molecules, depending on the flexibility of the linker,^[25]^ the presence of functional groups as well as the guest species.^[26]^ In Ga‐MIL‐53‐L^2^, a change in cell volume of 17.5 % is observed. Dehydration leads to a decrease of the unit cell volume from 1244.3(2) to 1027.47(5) Å^3^ (Figure 6, top). The pore diameter of Ga‐MIL‐53‐L^2^ decreases from Ga‐MIL‐53‐L^2^_lp1 to Ga‐MIL‐53‐L^2^_np by ca. 0.8 Å. Using the DFT optimized structure models, calculations with Zeo++ result in pore diameters (for the largest included sphere) of 3.5, 3.6 and 2.8 Å for Ga‐MIL‐53‐L^2^_lp1, Ga‐MIL‐53‐L^2^_lp2 and Ga‐MIL‐53‐L^2^_np, respectively.^[27]^

**Figure 7:**
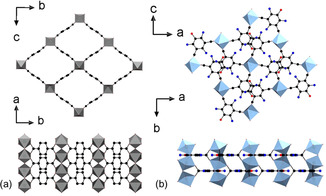
Comparison of the high temperature forms of Al‐MIL‐53‐BDC [Al(OH)(*p*‐BDC)] (a) and Ga‐MIL‐53‐L^2^_np (b) with linear and V‐shaped linker molecules, respectively. The different linker shapes lead to different pore structures (1D vs. 0D, top) and torsion angles between the benzene ring and the carboxylate groups (bottom). Al(OH)_2_O_4_‐polyhedra in grey, Ga(OH)_2_O_4_‐polyhedra in pale blue, oxygen in red, nitrogen in blue, carbon in black. Oxygen atoms of nitro groups have been omitted for clarity.

A comparison of the high temperatures phases of Al‐MIL‐53 [Al(OH)(*p*‐BDC)] and Ga‐MIL‐53‐L^2^ is shown in Figure 7. The linear linker in Al‐MIL‐53‐BDC leads to the open pore form with 1D pores while the V‐shaped linker results in the formation of 0D pores (cages) in Ga‐MIL‐53‐L^2^_np (Figure 6, bottom). In addition, differences in the torsion angle between the benzene ring and the carboxylate groups are clearly visible. In Al‐MIL‐53‐BDC the carboxylate groups and the benzene ring are in plane while they are rotated by 90° in the Ga compound.

The systematic synthetic studies on Ga‐MIL‐53‐L^2^ demonstrated the almost selective incorporation of linker L^2^ and the structure‐directing role of the nitro group located in the 2‐position. To confirm the structure directing role of the nitro group, 2‐nitro‐isophthalic acid (*m*‐H_2_BDC‐NO_2_, Figure S14) was employed. While reactions with Al(NO_3_)_3_ only led to X‐ray amorphous products, the use of Ga(NO_3_)_3_ resulted in the formation of the MIL‐53 type framework as observed in Ga‐MIL‐53‐L^2^. Details regarding the Rietveld refinement and crystal structure can be found in Table 1 and the Supporting Information (section 9).

### Thermal analysis

The thermal behaviour of the title compounds was also studied by thermogravimetric (TG) temperature dependent (TD) DRIFT measurements. According to the TG curve guest molecules in Al‐CAU‐10‐L^0, 2, 4, 6^ and the two Ga compounds Ga‐MIL‐53‐L^2^_lp1/_lp2 are desorbed up to 170 °C (Figures S15, S16, S17 and Table S2). A plateau is observed and the decomposition of the frameworks takes place above ca. 300 °C resulting in the formation of *ß*‐Ga_2_O_3_ or X‐ray amorphous Al_2_O_3_ (Figure S18).^[28]^ TD DRIFT measurements allow to follow the removal of incorporated guest molecules and changes of the local structure. The results for Al‐CAU‐10‐L^0, 2, 4, 6^ and Ga‐MIL‐53‐L^2^_lp1 are presented in Figure S19 and S20. Similar changes of band intensities are observed for both compounds. Water molecules are desorbed between 40 and 140 °C, which is clearly visible in the changes in band intensities and shape. At around 100 °C, the band of the ‐OH stretching vibration of the aromatic hydroxyl group at 3060 cm^−1^ becomes clearly visible. Simultaneously, at 2870 cm^−1^ a band of low intensity can be assigned to intramolecular H‐bonding between the hydroxyl and some nitro groups as evidenced in the DFT optimized geometries of the L^4^ linker (Figure S19 and S20, band 2 and band 3).^[29]^ After the decomposition of the compounds (*T*>360 °C) to their respective metal oxides, a prominent band of adsorbed CO is found at higher temperatures (Figure S19 and S20, band 4). The ability of metal oxides, such as Al_2_O_3_ or Ga_2_O_3_ to adsorb CO, leading to the characteristic vibrational band at 2250 cm^−1^, has been previously reported.^[30]^

### Sorption properties

**Figure 8:**
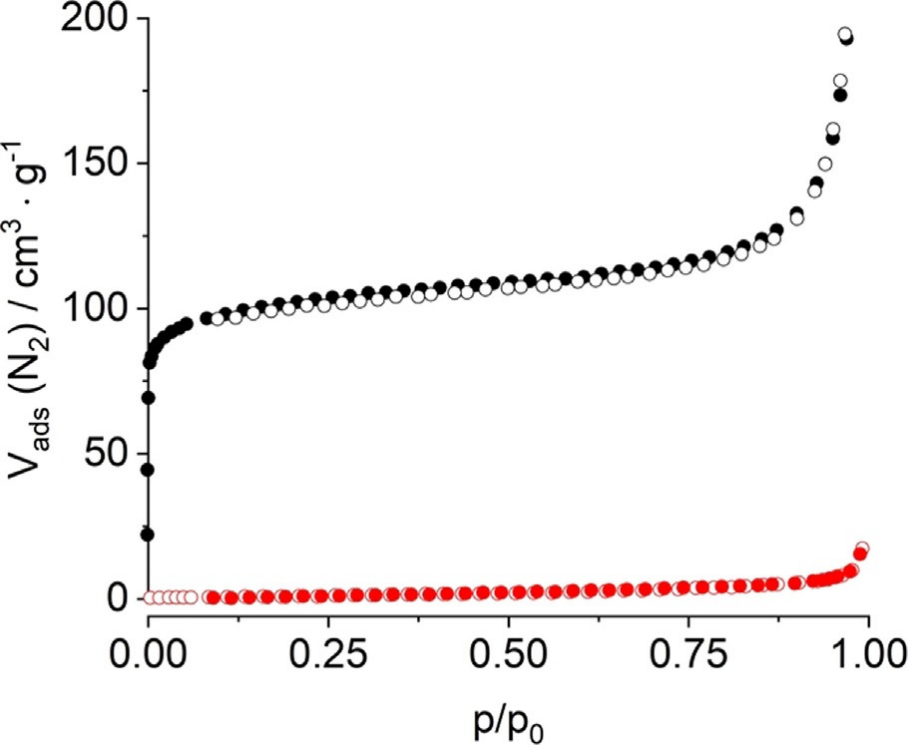
Nitrogen sorption isotherms of Al‐CAU‐10‐L^0, 2, 4, 6^ (black circles) and Ga‐MIL‐53‐L^2^ (red circles) measured at 77 K. Filled symbols represent adsorption, empty symbols represent desorption.

**Figure 9:**
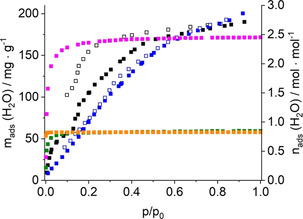
Experimental water sorption isotherms of Al‐CAU‐10‐L^0, 2, 4, 6^ (blue squares) and Ga‐MIL‐53‐L^2^ (black squares) measured at 298 K, together with simulated sorption isotherms for Ga‐MIL‐53‐L^2^_lp1 (pink squares), Ga‐MIL‐53‐L^2^_lp2 (green squares) and Ga‐MIL‐53‐L^2^_np (orange squares). Filled symbols represent adsorption, empty symbols represent desorption. One has to keep in mind that structural changes upon water adsorption are not taken into account in the GCMC calculations.

Compounds with MIL‐53 and CAU‐10 framework types are known to exhibit porosity with respect to N_2_ and H_2_O. Therefore, sorption experiments were conducted. Prior to the experiments, samples of Al‐CAU‐10‐L^0, 2, 4, 6^ and Ga‐MIL‐53‐L^2^_lp1 were activated under reduced pressure (*p*<10^−2^ mbar) at 180 °C for 16 h and 230 °C for 4 h, respectively. Nitrogen sorption isotherms of type I for Al‐CAU‐10‐L^0, 2, 4, 6^ and type III for Ga‐MIL‐53‐L^2^ were observed, indicating typical microporous and non‐porous sorption behaviour, respectively (Figure 8). For Al‐CAU‐10‐L^0, 2, 4, 6^ a BET surface area of *a*_sBET_=380 m^2^ g^−1^ and a micropore volume of *V*_mic_=0.17 cm^3^ g^−1^ were determined. Ga‐MIL‐53‐L^2^ does not show any uptake due to the small pores, that is, smaller than the kinetic diameter of N_2_, and weak host‐guest interactions. The experimental water adsorption isotherms of Ga‐MIL‐53‐L^2^ and the GCMC simulated ones are shown in Figure 9. They strongly deviate from the ones of CAU‐10‐H and Al‐MIL‐53.[^8^, ^31^] For Al‐CAU‐10‐L^0, 2, 4, 6^ a maximum water uptake of 200 mg g^−1^ is found. The typical S‐shape of the isotherm is not observed which is in line with the results of the structural studies since Al‐CAU‐10‐L^0, 2, 4, 6^ exhibits no structural flexibility.

The structural changes in Ga‐MIL‐53‐L^2^ are reflected in the shape of the water sorption isotherm. At low relative pressures, up to *p*/*p*_0_=0.17, one H_2_O molecule per formular unit is adsorbed which correlates well with the formation of Ga‐MIL‐53‐L^2^_lp2. The GCMC simulated water adsorption isotherms for Ga‐MIL‐53‐L^2^_lp2 and _np forms show nearly identical uptake of 60 mg g^−1^ at saturation, which matches the experimental uptake at the first plateau of the adsorption isotherm (Figure 9).

However, due to its more confined porosity, the np form saturates at slightly lower relative pressure than the Ga‐MIL‐53‐L^2^_lp2 structure. The steep adsorption profile for the Ga‐MIL‐53‐L^2^_lp2/_np is associated with a high water adsorption enthalpy of ≈60 kJ mol^−1^ and 70 kJ mol^−1^, respectively, which also emphasizes the high hydrophilicity of this MOF. Further, the GCMC calculated water saturation uptake of 180 mg g^−1^ of Ga‐MIL‐53‐L^2^_lp1 clearly manifests the experimentally observed water loading at higher relative pressure with a maximum uptake of 190 mg g^−1^. This whole set of simulations supports that there is a water‐induced structural transition from Ga‐MIL‐53‐L^2^_lp2 to _lp1 above *p*/*p*_0_=0.1.

GCMC derived preferential arrangements of the adsorbed water molecules within the pores of Ga‐MIL‐53‐L^2^_np and _lp1 are depicted in Figure S32 and Figure S33, respectively. In the case of the np form, μ‐OH sites are involved in intra‐framework O(μ‐OH)⋅⋅⋅O(OH) H‐bonds as H‐donors, and as H‐acceptors for the neighbouring adsorbed water molecules. The latter also form H‐bonds with ‐OH and ‐NO_2_ sites of the linker molecules. In Ga‐MIL‐53‐L^2^_lp1, at *p*/*p*_0_≈0.01 the μ‐OH sites only act as H‐donors to water molecules (Figure S33). The adsorbed water molecules form H‐bonds between each other and also interact with adjacent ‐OH and ‐NO_2_ sites of the linker molecules. This leads at *p*/*p*_0_≈0.1, to an extended H‐bonded 3D network (Figure S33).

## Conclusions

In conclusion, the in situ nitration of H_2_L^0^ by Al(NO_3_)_3_ and Ga(NO_3_)_3_ before framework formation, leads to linker molecules with nitro groups in the 2‐, 4‐ and 6‐positions, detected by ^1^H NMR spectroscopy. As proven by experiments using *m*‐H_2_BDC‐NO_2_, the nitro group's position at the aromatic ring strongly influences the formation of the final framework. A mixture of all observed linker molecules leads to a rigid Al‐CAU‐10, whereas the selective incorporation of the linker nitrated in position 2, gives rise to a flexible Ga‐MIL‐53 type compound as illustrated by the experimental and simulated water adsorption isotherms. This work highlights the very strong structure‐directing role of differently substituted linker molecules in the crystallization of Al and Ga‐MOFs, in this particular case leading to a new and uncommon MIL‐53 type structure with a bent linker molecule.

## Experimental Section

**Materials and methods**: Gallium nitrate heptahydrate (Ga(NO_3_)_3_ **⋅**7 H_2_O, ABCR, 99.99 % puratrem), aluminium nitrate nonahydrate (Al(NO_3_)_3_ **⋅**9 H_2_O, Grüssing, reinst), 5‐hydroxybenzene‐1,3‐dicarboxylic acid (H_2_L^0^, Sigma–Aldrich, >95 %), 2‐nitrobenzene‐1,3‐dicarboxylic acid (*m*‐H_2_BDC‐NO_2_, ABCR, >95 %) and glacial acetic acid (Grüssing, 99 %) were commercially obtained and used without further purification.

Powder X‐ray diffraction data (PXRD) were collected on a Stoe Stadi MP equipped with a MYTHEN 1 K detector (Cu${}_{K{}_{\alpha 1}}$ radiation, *λ*=1.5406 Å). Three‐dimensional electron diffraction data were collected on a JEOL JEM2100 TEM, equipped with a Timepix detector from Amsterdam Scientific Instruments. Infrared (IR) spectra were measured on a Bruker ALPHA‐FT‐IR A220/d‐01 using an ATR‐unit. ^1^H NMR spectra were recorded with a Bruker AVANCE III HD Pulse Fourier Transform spectrometer equipped with a cryo‐probehead Prodigy BBO400S1 BB‐H&F‐d‐05‐Z operating at a frequency of 400.13 MHz (^1^H). ^1^H ^13^C HMBC NMR spectra were recorded with a Bruker AvanceNEO 500 operating at a frequency of 500.13 MHz (^1^H) and 125.76 MHz (^13^C). Referencing was performed using deuterium oxide/ sodium deuteroxide (1.25 %). The CHNS‐measurements were performed with a vario MICRO cube elemental analyser from Elementar. Thermogravimetric data was collected on a NETZSCH STA 409 CD analyser (airflow=7.5 dm^3^ h^−1^, heating rate=4 K min^−1^) and on a Linseis STA PT 1000 (airflow=6 dm^3^ h^−1^, heating rate=4 K min^−1^). Sorption measurements were carried out using a BEL Japan Inc. BELSORP‐max with nitrogen gas and water vapour at 77 and 298 K, respectively. Depending on the compound, the samples were treated for 16 h at a temperature between 180 and 240 °C under reduced pressure (*p*<10^−2^ mbar) prior to the measurement. The syntheses of the compounds were carried out in Pyrex® glass vials (*V=*6 mL) which were heated in aluminium blocks or in regular ventilation ovens using custom‐made steel autoclaves with Teflon® inserts (total volume of 2 mL).^[32]^ Temperature dependent PXRD data (TD PXRD) were collected with a Stoe capillary furnace in 0.5 mm quartz capillaries. Temperature dependent DRIFT (TD DRIFT) measurements were conducted on a Bruker Vertex70 FTIR‐spectrometer using a Praying Mantis™ diffuse reflection accessory and a Praying Mantis™ High Temp. Reaction Chamber by Harrick scientific products. More details to the experimental methods are given in the Supporting Information. In situ IR experiments were carried out employing the ATR‐IR unit ReactIR 45 m by Mettler Toledo with an AgX‐Fiber 6.5 mm probe.

**Syntheses**: For the synthesis of Al‐CAU‐10‐L^0, 2, 4, 6^ 56.4 mg H_2_L^0^, 500 μL deionized water, 300 μL acetic acid and 200 μL of an aqueous solution of aluminium nitrate nonahydrate (1 mol L^−1^) were mixed in a 6 mL Pyrex® glass vial under stirring for 30 seconds at maximum rate (Table 2). The suspension was heated in an aluminium block for 1 h at 120 °C and after cooling to room temperature the yellow product was separated by centrifugation in a 3 mL vial at 9000 rpm for 3 min. Residues of H_2_L^0^ were removed by washing two times with methanol (redispersion and centrifugation). The yellowish solid was dried at 80 °C for 1 h. [Al(OH)(C_8_H_2.08_O_5_(NO_2_)_0.92_)]**⋅**H_2_O **CHNS**: C=34.2 % (34.0 % calcd), H=2.7 % (1.8 % calcd) and 4.1 % (calcd 4.6 %).

**Table 2:** Overview of reaction parameters for the synthesis of Al‐CAU‐10‐L^0, 2, 4, 6^, Ga‐MIL‐53‐L2_lp1, Ga‐MIL‐53‐L2_lp2, Ga‐MIL‐53‐L2_np as well as Ga‐MIL‐53‐m‐BDC‐NO2.

Compound	M:L	AA : H_2_O	*T*,*t* [°C,h]	Yield [%]
Al‐CAU‐10‐L^0, 2, 4, 6^	1:1.5	1:2.4	120, 1	63.5
Ga‐MIL‐53‐L^2^_lp1	1:2.1	1:2.4	120, 24	28.9
Ga‐MIL‐53‐L^2^_p2	thermal activation			
Ga‐MIL‐53‐L^2^_np	thermal activation			
Ga‐MIL‐53‐*m‐*BDC‐NO_2_	1:1	–	120, 1	34.8

For the synthesis of Ga‐MIL‐53‐L^2^_lp1 56.4 mg H_2_L^0^, 500 μL deionized water, 300 μL acetic acid and 200 μL of an aqueous solution of gallium nitrate heptahydrate (0.7 mol L^−1^) were mixed in a 2 mL Teflon reactor, which was placed in a custom‐made steel autoclave and heated at 120 °C for 24 h. After isolation by centrifugation at 9000 rpm for 3 min, the samples were dried under atmospheric conditions. The second large pore form Ga‐MIL‐53‐L^2^_lp2 and the narrow pore form Ga‐MIL‐53‐L^2^_np were obtained by thermal treatment of Ga‐MIL‐53‐L^2^_lp1 at 130 °C and 240 °C in glass capillaries under reduced pressure (*p*<10^−2^ mbar), respectively. [Ga(OH)(C_8_H_2_O_7_N)]**⋅**3 H_2_O **CHNS**: C=26.7 % (26.3 % calcd), H=2.8 % (2.5 % calcd) and 3.3 % (calcd 3.8 %).

The linker 2‐nitrobenzene‐1,3‐dicarboxylic acid (*m*‐H_2_BDC‐NO_2_) was employed in the synthesis of Ga‐MIL‐53‐*m*‐BDC‐NO_2_. The compound is obtained by mixing 20.0 mg *m*‐H_2_BDC‐NO_2_, 900 μL deionized water, 50 μL NaOH (2 mol L^−1^) and 50 μL of an aqueous solution of gallium nitrate heptahydrate (0.72 mol L^−1^) in a 6 mL Pyrex® glass vial under stirring for 30 seconds at maximum rate. The suspension was heated in an aluminium block for 1 h at 120 °C and after cooling to room temperature the white product was separated by centrifugation in a 3 mL vial at 9000 rpm for 3 min. Remaining residues of *m*‐H_2_BDC‐NO_2_ were removed by washing two times with methanol (redispersion and centrifugation). The white solid was dried at 80 °C for 1 h. For characterization by PXRD the dry product was transferred into a capillary, which was sealed after activation of the product at 220 °C for 1 h under reduced pressure (*p*<10^−2^ mbar). [Ga(OH)(C_8_H_3_NO_6_)]**⋅**H_2_O **CHNS**: C=30.7 % (30.6 % calcd), H=2.4 % (1.9 % calcd) and 4.3 % (calcd 4.5 %).

**Structure solution and refinement**: All compounds were obtained as microcrystalline powders. Therefore PXRD data had to be used for the structure determinations. Crystal data and the results of the Rietveld refinements of Al‐CAU‐10‐L^0, 2, 4, 6^, Ga‐MIL‐53‐L^2^_lp2, Ga‐MIL‐53‐L^2^_np, Ga‐MIL‐53‐*m*‐BDC‐NO_2_ as well as the results of the Le Bail fit of Ga‐MIL‐53‐L^2^_lp1 are summarized in Table 1. The final Rietveld plots of Al‐CAU‐10‐L^0, 2, 4, 6^ and Ga‐MIL‐53‐L^2^_np are shown in Figure 1, the other plots are given in Figure S34, S37 and S39. More details on the structure determination can also be found in the Supporting Information (Figure S34–S46 and Table S8–S13). For the structure elucidation of Al‐CAU‐10‐L^0, 2, 4, 6^ the crystal data of CAU‐10‐CH_3_ ^[8]^ was used to create a starting model, which was refined by the Rietveld method^[33]^ using TOPAS Academic.^[34]^ The structure of Ga‐MIL‐53‐L^2^_np was solved from 3D electron diffraction (3D ED) data of a sub‐micron sized single crystal (Figure S36) and the structural model was subsequently refined against PXRD data. Details on the data‐collection procedure can be found in the Supporting Information. The structural information of Ga‐MIL‐53‐L^2^_np was used to create an initial model for Ga‐MIL‐53‐L^2^_lp2 and Ga‐MIL‐53‐*m*‐BDC‐NO_2_, which were also refined using the Rietveld method. For Ga‐MIL‐53‐L^2^_lp1 only a structureless Le Bail fit^[35]^ was carried out and the phase purity was confirmed (Figure S41). It is presumed water molecules occupying the pores of Ga‐MIL‐53‐L^2^ lp1 are arranged in a disordered fashion.

Deposition numbers 2057515, 2057516, 2057517, and 2057518 contain the supplementary crystallographic data for Al‐CAU‐10‐L^0, 2, 4, 6^, Ga‐MIL‐53‐L^2^_np, Ga‐MIL‐53‐*m*‐BDC‐NO_2_ and Ga‐MIL‐53‐L^2^_lp2, respectively. These data are provided free of charge by the joint Cambridge Crystallographic Data Centre and Fachinformationszentrum Karlsruhe Access Structures service www.ccdc.cam.ac.uk/structures.

**Molecular simulations**: With the purpose of checking the favourable stability of Ga‐MIL‐53 and Ga‐CAU‐10 topologies in terms of ground state electronic energies, we have thoroughly optimized L^2^ and L^4^ variants of the theoretical Ga‐CAU‐10 models using the CP2K program.^[36]^ The relevant DFT optimized geometries of Ga‐MIL‐53 systems are provided as supporting information. GCMC simulations were further performed to predict the water adsorption isotherms for Ga‐MIL‐53‐L^2^_lp2, Ga‐MIL‐53‐L^2^_lp1 and Ga‐MIL‐53‐L^2^_np crystal structures. All computational details are given in section 3 of the Supporting Information.

## Conflict of interest

The authors declare no conflict of interest.

## Supporting information

As a service to our authors and readers, this journal provides supporting information supplied by the authors. Such materials are peer reviewed and may be re‐organized for online delivery, but are not copy‐edited or typeset. Technical support issues arising from supporting information (other than missing files) should be addressed to the authors.

Supplementary
