# Supplementary material for: Metal‐Dependent and Selective Crystallization of CAU‐10 and MIL‐53 Frameworks through Linker Nitration
Source: Chemistry. 2021 Mar 10;27(28):7696–703. doi: 10.1002/chem.202100373 (PMC8252442; doi:10.1002/chem.202100373)
Supplement: Supplementary file 1 — Supplementary [file CHEM-27-7696-s001.pdf]

Chemistry–A European Journal

Supporting Information

Metal-Dependent and Selective Crystallization of CAU-10 and MIL-53 Frameworks through Linker Nitration

Timo Rabe,[a] Erik Svensson Grape,[b] Tobias A. Engesser,[a] A. Ken Inge,[b] Jonas Ströh,[a] Gitta Kohlmeyer-Yilmaz,[c] Mohammad Wahiduzzaman,[d] Guillaume Maurin,[d] Frank D. Sönnichsen,[c] and Norbert Stock*[a]

1. Set up and results of the combined in situ IR and optical light scattering experiments 2
2. ^{1}H-NMR-spectroscopy 4
3. Computational Details 8
4. Temperature dependent PXRD measurements, details on the structural flexibility and Structure of 2-nitro-benzene-1,3-dicarboxylic acid 9
5. Thermogravimetric measurements, DTA and CHNS analysis 12
6. IR-spectroscopy 15
7. Sorption Isotherms 19
8. Thermogravimetric measurement of $Ga(NO_3)_3 \cdot x\ H_2O$ 23
9. GCMC preferential arrangements of the adsorbed water 25
10. 3DED data, Rietveld refinements, Le Bail fit, asymmetric units and bond lengths 26
11. References 38

1. Set up and results of the combined in situ IR and optical light scattering experiments

The reaction of Al-CAU-10-$L^{0,2,4,6}$ was studied by *in situ* IR spectroscopy to investigate if 5-hydroxyisophthalic acid is nitrated before or after framework crystallization. Therefore the setup in Figure S1 was used. The IR-probe (**1**) was inserted into the reaction vessel (**2**) and placed in a transparent silicon oil bath (**3**) on a combined magnet stirrer and heater (**4**). Temperature monitoring of the oil bath was acchived by a digital thermometer (**5**) and a laser pointer (**6**) was employed to trigger the Faraday-Tyndall-effect from small particles in solution. Observations were recorded with a smart phone video camera (**7**) and experimental data was accumulated, processed and stored on the computer (**8**). The IR spectra were recorded every 10 s for 1h. The optical progress of the reaction is displayed in Fig. S2, the IR spectra for Al-CAU-10-$L^{0,2,4,6}$ and Ga-MIL-53-L^{2} after 20s, 1min 20 s, 2 min, 5 min and 10 min are shown in Fig. S3 and S4.

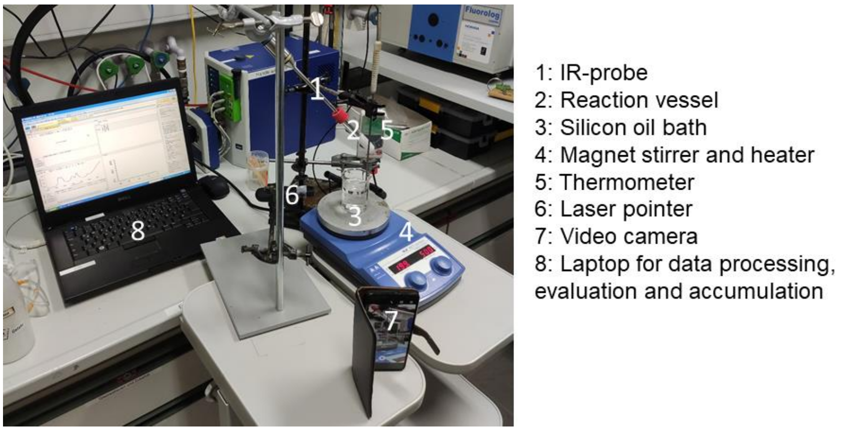

Figure S1: Setup of the in situ IR experiments with (1) IR-probe, (2) reaction vessel, (3) silicon oil bath, (4) magnet stirrer and heater, (5) thermometer, (6) laser pointer, (7) video camera, (8) laptop for data processing, evaluation and accumulation.

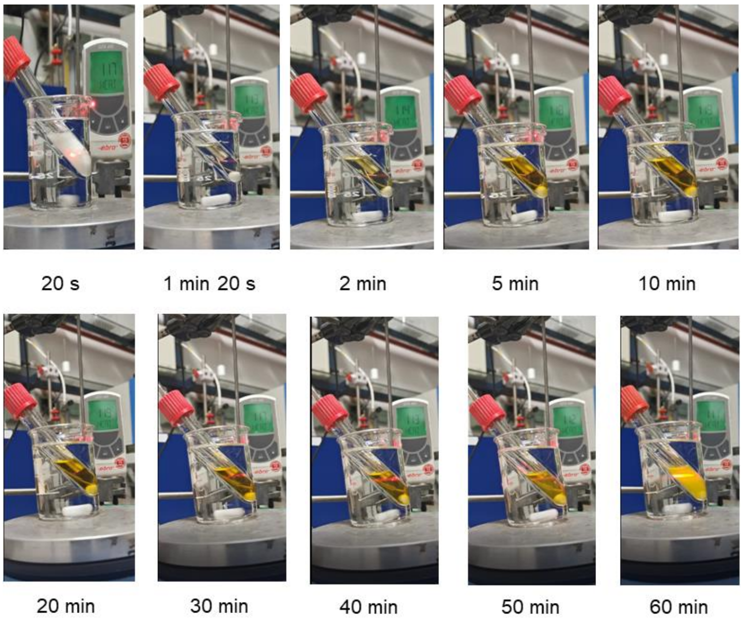

Figure S2: Pictures of the reaction mixture of Al-CAU-10-$L^{0,2,4,6}$ during the in situ IR measurement with the setup described in Figure S13 after 20 s, 1 min 20 s, 2 min, 5 min , 10 min, 20 min, 30 min, 40 min, 50 min and 60 min.

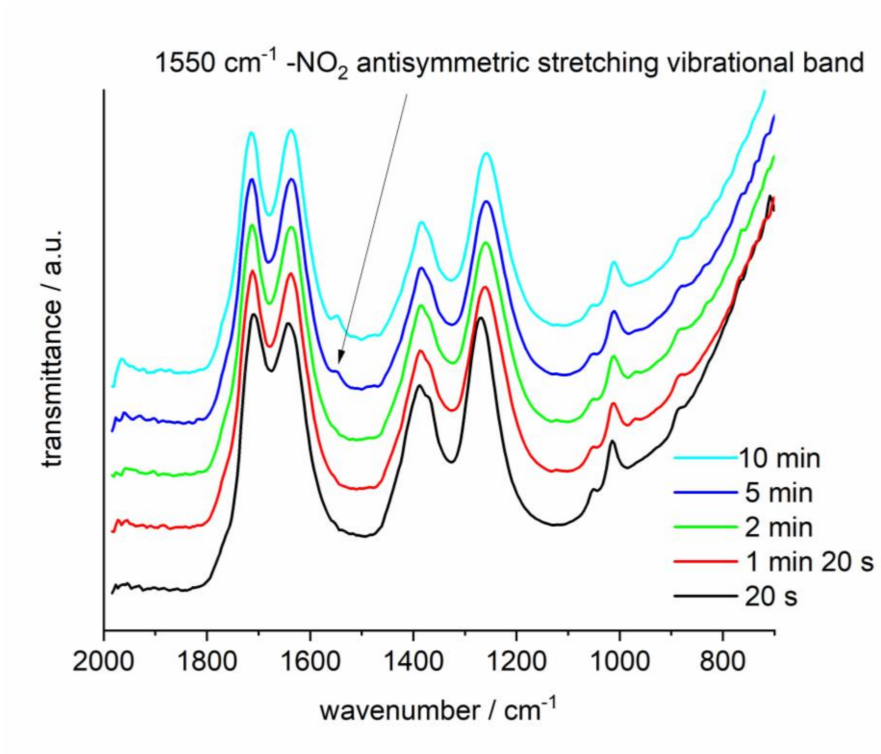

Figure S3: In situ IR spectra of the reaction mixture of Al-CAU-10-$L^{0,2,4,6}$ after 20 s, 1 min 20 s, 2 min, 5 min and 10 min with the vibrational band for the -NO_2 antisymmetric stretching vibration of aromatic nitro compounds at 1550 cm^{-1}.

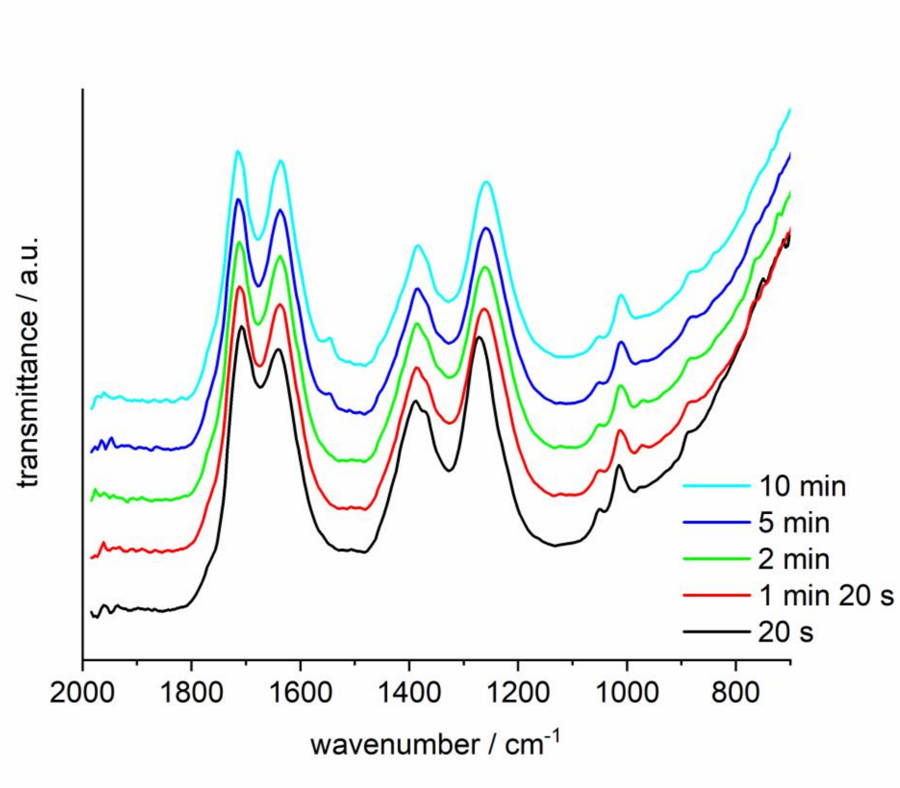

Figure S4: In situ IR spectra of the reaction mixture of Ga-CAU-10-L^2 after 20 s, 1 min 20 s, 2 min, 5 min and 10 min with the vibrational band for the -NO_2 antisymmetric stretching vibration of aromatic nitro compounds at 1550 cm^{-1}.

2. ^{1}H-NMR-spectroscopy

Al-CAU-10-L^{0,2,4,6} and Ga-MIL-53-L^2

^{1}H-NMR spectra were recorded with a Bruker AVANCE III HD Pulse Fourier Transform spectrometer equipped with a cryo-probehead Prodigy BBO400S1 BB-H&F-D-05-Z operating at a frequency of 400.13 MHz (1H). Referencing was performed using deuterium oxide/ sodium deuteroxide (1.25 %). HMBC-NMR-spectra were recorded with a Bruker Bruker AvanceNeo 500 operating at a frequency of 500.13 MHz (^{1}H) and 125.76 MHz (^{13}C).

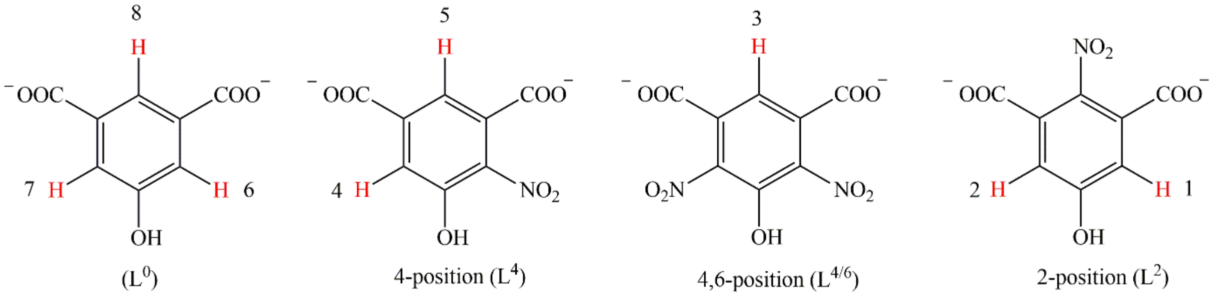

Figure S5: Labeling of the hydrogen atoms in the linker molecules of Al-CAU-10-L^{0,2,4,6}, Ga-MIL-53-L^2_lp1 and Ga-MIL-53-L^2_lp2.

Figure S6 shows the ^{1}H-NMR spectra of dissolved Al-CAU-10-L^{0,2,4,6}, Ga-MIL-53-L^2_lp1 with different concentrations of the ligands L^0, L^4, L$^{4/6}$ and L^2 and Fig. S7 the ^{1}H-^{13}C HMBC spectra of Ga-MIL-53-L^2.

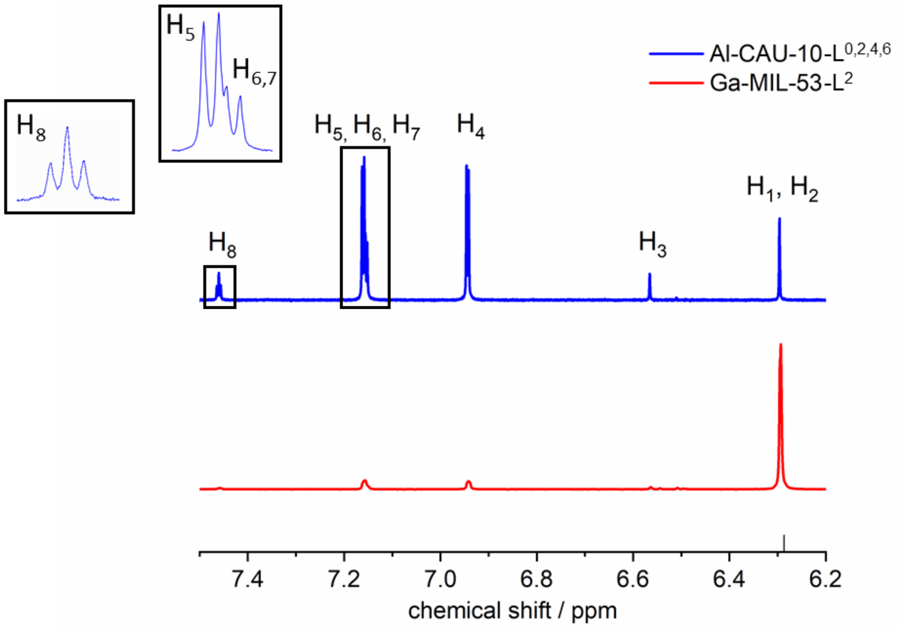

Figure S6: ^{1}H-NMR spectra of Al-CAU-10-L^{0,2,4,6} and Ga-MIL-53-L^2 (500MHz, NaOD/D_2O, 298K).

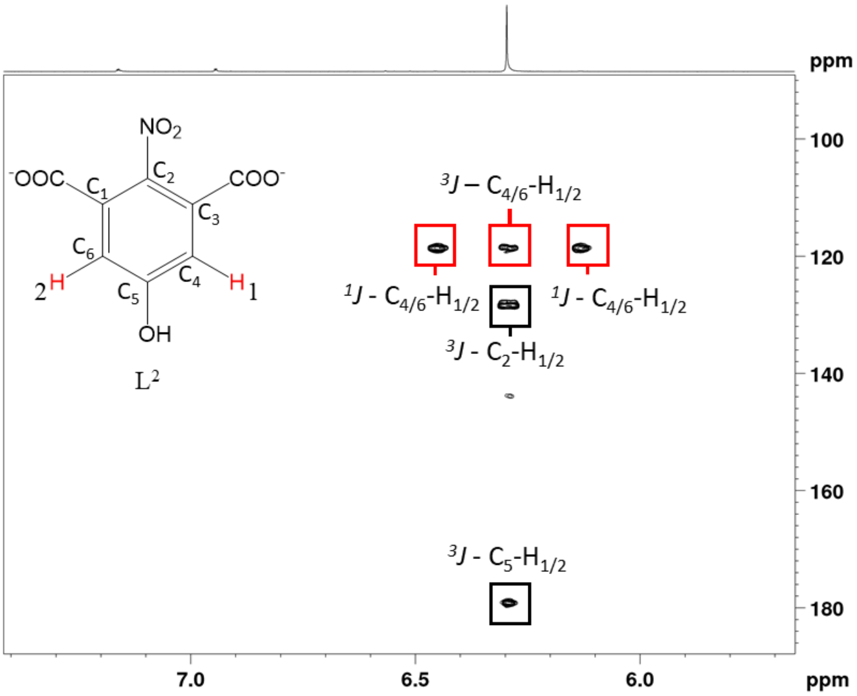

Figure S7: ^{1}H-^{13}C HMBC spectra of Ga-MIL-53-L^2 (500 MHz/125 MHz, NaOD/D_2O 1.25 %, 298 K).

The assignment of the signals in the high field region of the NMR spectra to the different functionalized linker molecules is given in Figure S5. To unequivocally assign the signals at 6.57 and 6.30 ppm to the molecules L$^{4/6}$ and L^2 additionally ^{1}H-^{13}C HMBC-NMR spectra were recorded (Fig. S7). The highlighted crosspeaks show the expected pattern for two chemically equivalent aromatic protons in meta-position. In the center of the one bond coupling cross peak, which is split by the ^1J-coupling constant into a doublet in the F2-dimension of the undecoupled HMQC experiment, another cross peak is observed between the respective proton and the chemically equivalent, symmetry related carbon via the much smaller ^3J-coupling constant.

To determine the molar ratios of linker molecules in the MOFs, the aromatic signals were normalized to the number of H-atoms in the respective linker and the integrals were used for the calculations.

Al-CAU-10-L^{0,2,4,6}:

^{1}H-NMR (400 MHz, NaOD, D_2O, 298 K) δ (ppm): L^0: 7.46 (t, 1H, H_8); 7.15 (d, 2H, $H_{6,7}$); L^4 7.16 (d, 1H, H_5); 6.94 (d, 1H, H_4); L$^{4/6}$ 6.57 (s, 1H, H_3); L^2 6.30 (s, 2H, $H_{1,2}$), ppm.

Total amount in Al-CAU-10-L^{0,2,4,6}: L^0 15 %, L^4 71 %, L$^{4/6}$ 7 %, L^2 7 %.

Ga-MIL-53-L^2:

^{1}H-NMR (400 MHz, NaOD, D_2O, 298 K) δ (ppm): L^4 7.16 (d, 1H, H_5); 6.94 (d, 1H, H_4); L^2 6.30 (s, 2H, $H_{1,2}$), ppm.

Total amount in Ga-MIL-53-L^2: L^4 8 %, L^2 92 %.

Experiments with KNO_3 and H_2L_0 under the reaction conditions for Al-CAU-10-L^{0,2,4,6} lead to the same nitration products formed with the metal nitrates $Al(NO_3)_3$ and $Ga(NO_3)_3$. No solid formed during these reactions. In order to conduct ^{1}H-NMR spectroscopic measurements the reaction solution was evaporated and the remaining solid dissolved in NaOD/D_2O.

Al-CAU-10-L^{0,2,4,6} influence of the reaction time

No significant differences of the signals integrals were observed, after 1 h and 24 h reaction time, in both ^{1}H-NMR spectra of samples (Fig. S8).

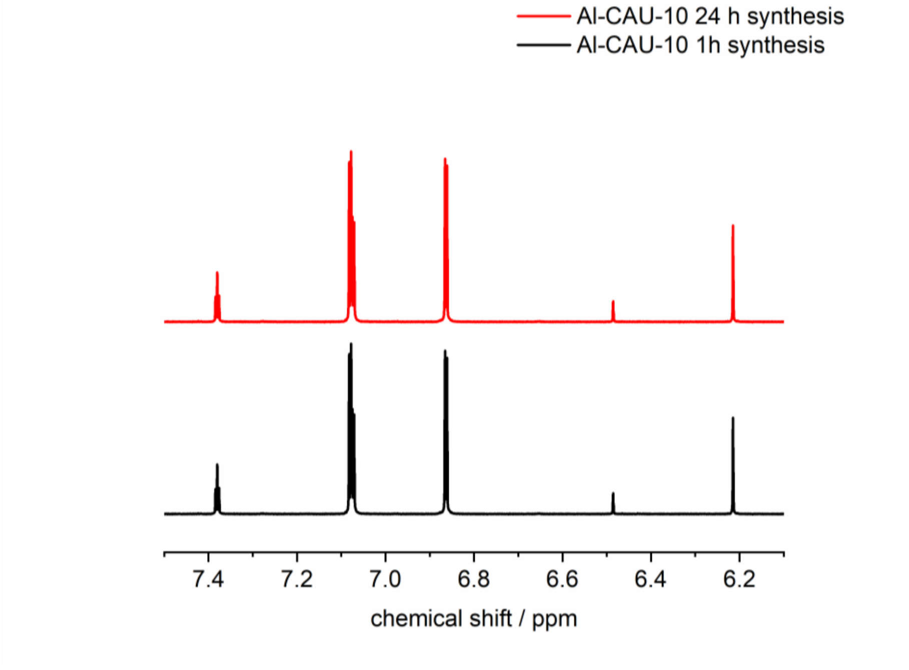

Figure S8: ^{1}H-NMR spectra of the dissolved Al-CAU-10-L^{0,2,4,6} after 1 h and 24 h reaction time.

Ga-MIL-53-L^2 influence of the reaction time

Three reactions were carried out and the products were isolated after a reaction time of 1 h, 2h and 24h, respectively. The samples were dissolved and the molar ratios of the linker molecules in the samples were determined by NMR-spectroscopy. The amount of L^2 increases in the samples with reaction time (Fig. S9, Tab. S1).

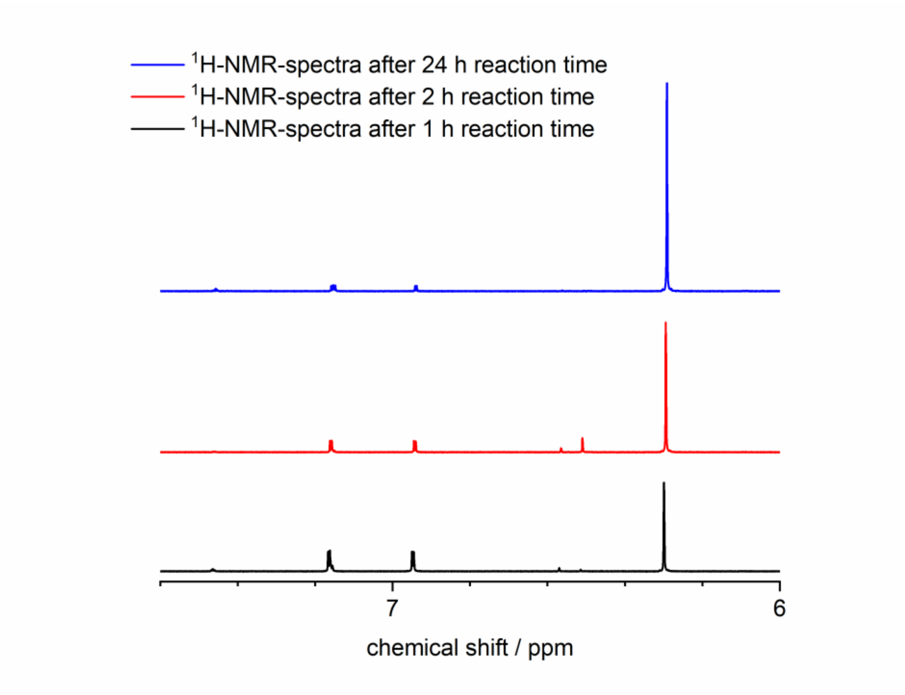

Figure S9: ^{1}H-NMR spectra of the dissolved Ga-MIL-53-L^2_lp1 with a reaction time of 1h and 2 h in comparison with 24 h.

Table S1: Summary of the experimental results of ^{1}H-NMR spectroscopic measurements. The linker content of L^0, L^2, L^4, $L^{4/6}$ of Ga-MIL-53-L^2 after a reaction time of 1, 2 and 24 h is given.

**Reaction time / h**	**1**	**2**	**24**
**Product**		**content / %**	
**L^0**	6	0	0
**L^2**	47	78	92
**L^4**	43	16	8
**$L^{4/6}$**	4	6	0

3. Computational Details

DFT calculations

DFT geometry optimization calculations of all the structures mentioned in the manuscript were performed with the Quickstep module[1] of the CP2K program[2,3] which is based on Gaussian Plane Wave (GPW) formalism. We have employed the general gradient approximation (GGA) to the exchange-correlation functional according to Perdew-Burke-Ernzerhof (PBE)[4] in a combination of Grimme's DFTD3 semi-empirical dispersion corrections.[5,6] Triple-ζ plus valence polarized Gaussian-type basis sets (TZVP-MOLOPT) were considered for all atoms, except for the Al and Ga centers, where short ranged double-ζ plus valence polarization functions (DZVP-MOLOPT) were employed.[7] The interactions between core electrons and valence shells of the atoms were described by the pseudo-potentials derived by Goedecker, Teter, and Hutter (GTH).[8-10] The auxiliary plane wave basis sets were truncated at 400 Ry. In addition, single point energy calculations were performed to extract the atomic partial charges of Ga-MIL-53 systems applying the REPEAT fitting strategy for the periodic system as implemented in the CP2K code.

Water Adsorption Simulations

The DFT geometry optimized model of Ga-MIL-53 systems in lp1, lp2 and np form with L^2 linker were further employed in Grand Canonical Monte Carlo (GCMC) calculations to determine the water adsorption properties of these Ga-MIL-53 forms at 298 K using the RASPA code.[11] We considered a simulation box of 18 conventional unit cells (2×5×3) of the systems maintaining atoms at their initial positions. The water molecules were described by the TIP4P-Ew potential model corresponding to a microscopic representation of four LJ sites.[12] The interactions between the guest water molecules and the MOF structure were described by a combination of site-to-site Lennard-Jones (LJ) contributions and Coulombic terms. Universal force field (UFF)[13] parameters were adopted to describe the LJ parameters for the atoms in the framework, respectively. However, following the treatment adopted in other well-known force fields,[14,15] the hydrogen atoms of the μ_2-OH moieties and –OH of the organic linkers as well as Ga atoms are allowed to interact with the adsorbate water molecules via the coulombic potential only as justified in previous studies on similar MOF topologies.[16] Short-range dispersion forces were truncated at a cutoff radius of 12 Å while the interactions between unlike force field centers were treated by means of the Lorentz-Berthelot combination rule. The long-range electrostatic interactions were handled using the Ewald summation technique. For each point in the adsorption isotherm, a typical 1×10^5 MC steps for equilibration and 2×10^5 MC steps have been used for production runs. The adsorption enthalpy at low coverage (Δh) was calculated through the configurational-bias Monte Carlo simulations performed in the NVT ensemble using the revised Widom's test particle insertion method.[17]

4. Temperature dependent PXRD measurements, details on the structural flexibility and Structure of 2-nitro-benzene-1,3-dicarboxylic acid

Temperature dependent PXRD measurements are shown below (Fig. S10-S11).

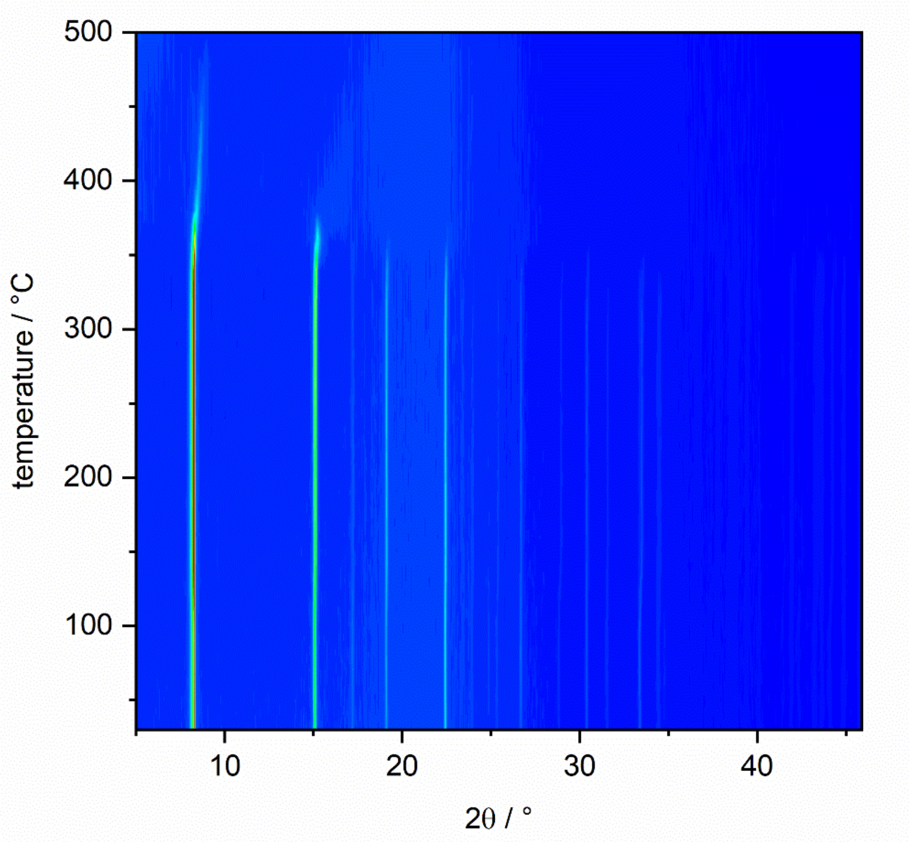

Figure S10: Temperature dependent PXRD measurement of Al-CAU-10-L^{0,2,4,6}.

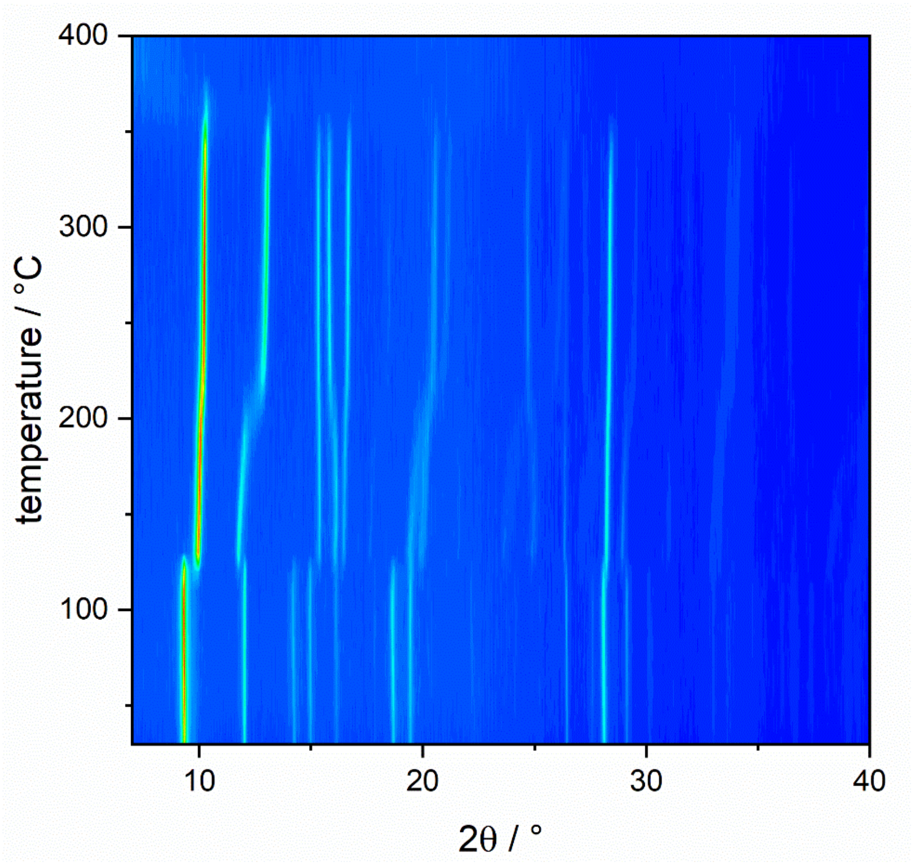

Figure S11: Temperature dependent PXRD measurement of Ga-MIL-53-L^2_lp1.

Deviations of the decomposition temperatures obtained from temperature dependent PXRD measurements and thermogravimetric measurements can be explained through the different experimental setups. Whereas the thermogravimetric measurements are performed in a flow of air in Al_2O_3 crucibles, the temperature dependent PXRD measurements are carried out in densely packed 0.5 mm quartz capillaries.

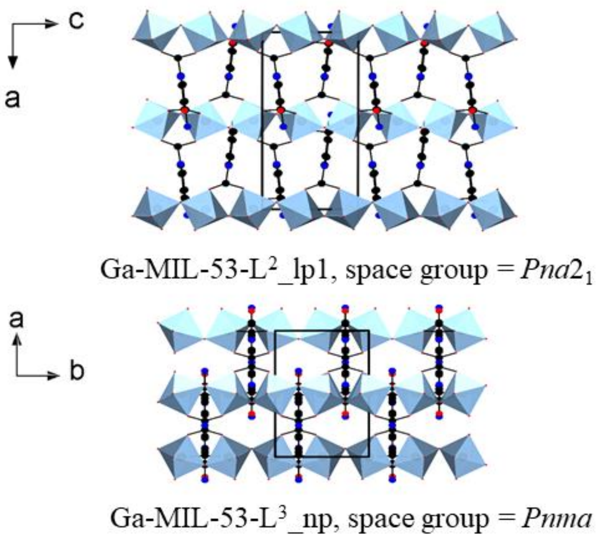

Figure S12. Comparison of the crystal structures of Ga-MIL-53-L2_lp1 (top) and Ga-MIL-53-L2_np (bottom).

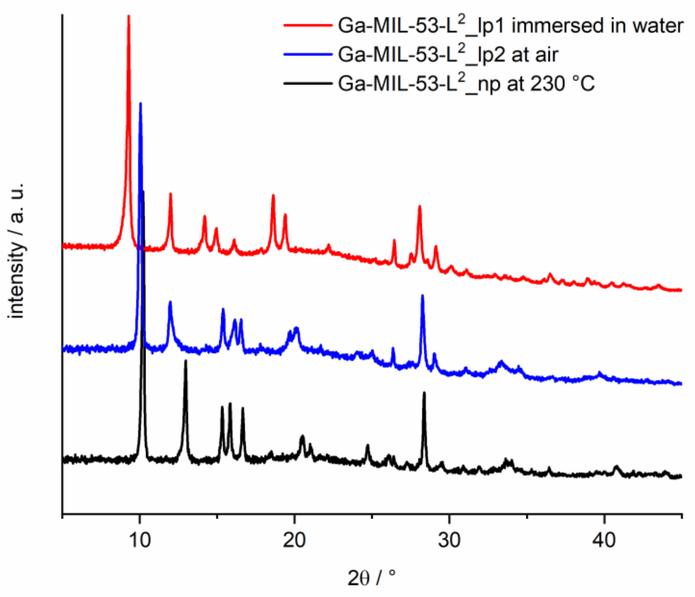

Figure S13: PXRD patterns of Ga-MIL-53-L^2_np (black, activated at 230 °C), Ga-MIL-53-L^2_lp2 (blue, at air) and of activated Ga-MIL-53-L^2_lp1 (green, immersed in H_2O).

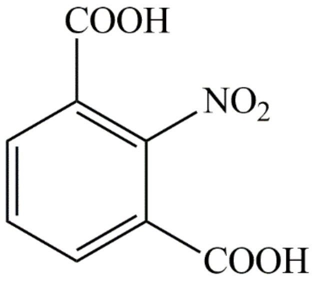

Figure S14: Structure of *m*-H_2BDC-NO_2.

5. Thermogravimetric measurements, DTA and CHNS analysis

The thermogravimetric measurements were performed on a NETZSCH STA 409 CD analyzer (airflow = 7.5 dm^3 h^{-1}heating rate = 4 K/min) and Linseis STA PT 1000 (nitrogen flow = 6 dm^3 h^{-1}, heating rate = 4 K min^{-1}). The sample amount was approximately 30 mg for each sample. PXRD patterns collected after the measurement are shown below (Fig. S18) along with the plots of the thermogravimetric measurements (Fig. S15-17). PXRD analysis of the residues obtained from Al-CAU-10-$L^{0,2,4,6}$ after the TG measurements showed the formation of an amorphous decomposition product. Theoretical calculations were done assuming the residue was Al_2O_3.

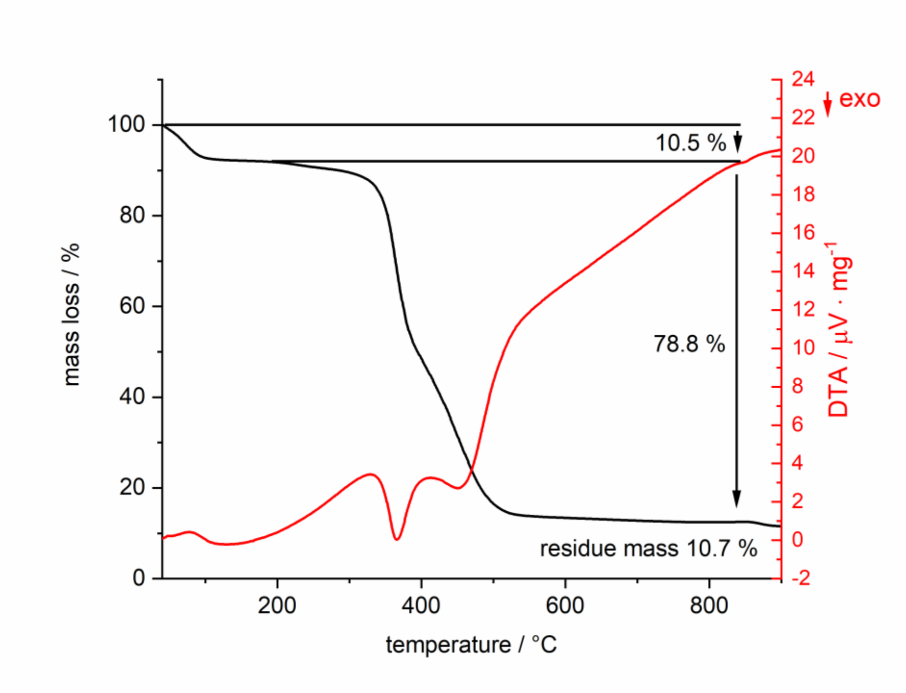

Figure S15: Thermogravimetric (black) and DTA (red) curve of Al-CAU-10-$L^{0,2,4,6}$.

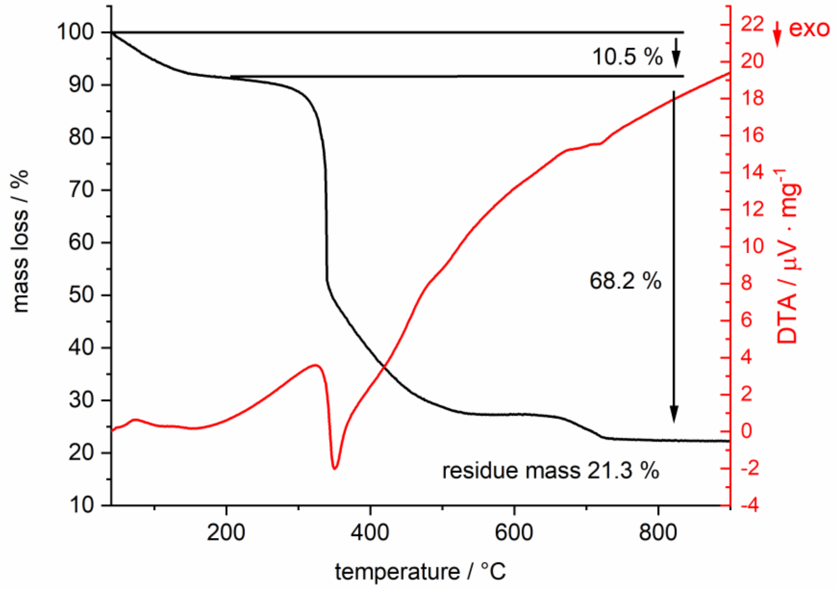

Figure S16: Thermogravimetric (black) and DTA (red) curve of Ga-MIL-53-L^2_lp1.

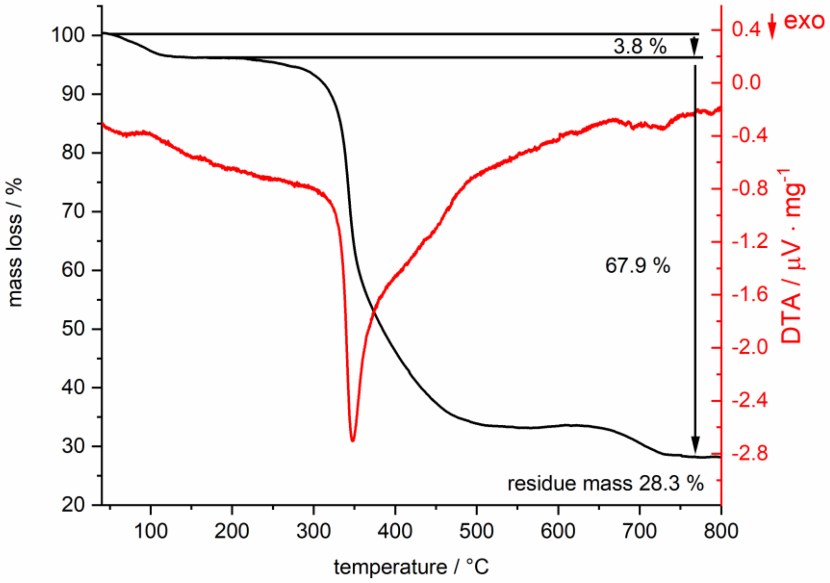

Figure S17: Thermogravimetric (black) and DTA (red) curve of Ga-MIL-53-L^2_lp2.

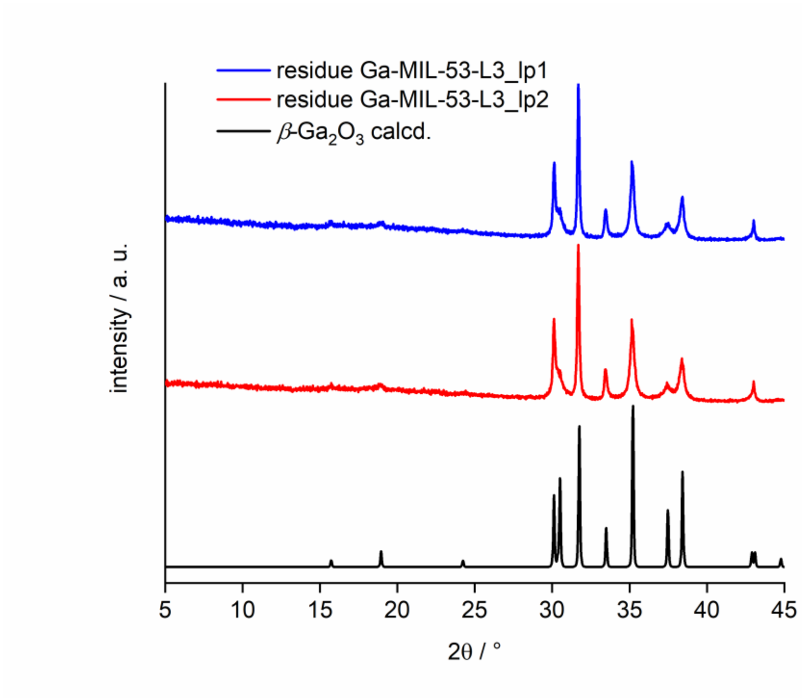

Figure S18: PXRD pattern Ga-MIL-53-L^2_lp1 and Ga-MIL-53-L^2_lp2s residues after the thermogravimetric measurement.[1]

The following compositions of the title compounds were used for the evaluation of the TG curves.

TG: Al-CAU-10-$L^{0,2,4,6}$ $[Al(OH)(C_8H_{2.08}O_5(NO_2)_{0.92})] \cdot 3\ H_2O \cdot 3\ CH_3COOH$

TG: Ga-MIL-53-L^2_lp1 $[Ga(OH)(C_8H_2O_7N)] \cdot 2.5\ H_2O \cdot 1.5\ CH_3COOH$

TG: Ga-MIL-53-L^2_lp2 $[Ga(OH)(C_8H_2O_7N)] \cdot H_2O$

Table S2: Results of the thermogravimetric analysis for Al-CAU-10-$L^{0,2,4,6}$, Ga-MIL-53-L^2_lp1 and Ga-MIL-53-L^2_lp2 at air.

**Step**	**Solvent / wt%**			**Ligand / wt%**			**Residue mass / wt%**		
**Product**	Obs	Calcd	ΔT/°C	Obs	Calcd	ΔT/°C	Obs	Calcd	ΔT/°C
**Al-CAU-10-L2**	10.5	10.8	25-270	78.8	78.9	270-880	10.7	10.2	880-1000
**Ga-MIL-53-L3_lp1**	10.5	10.1	25-240	68.2	68.9	240-740	21.3	21.0	740-1000
**Ga-MIL-53-L3_lp2**	3.8	5.5	25-240	67.9	67.9	240-740	28.3	29.3	740-1000

6. IR-spectroscopy

The temperature dependent FT-IR measurements are shown in Fig. S19-S20. For the temperature dependent FT-IR measurements, the microcrystalline samples of Al-CAU-10-$L^{0,2,4,6}$ and Ga-MIL-53-L^2_lp1 were homogenized with KBr and measured in a temperature range of 20-520 °C in air. The evaluation of characteristic bands marked with numbers 1 to 4 is given in the main manuscript.

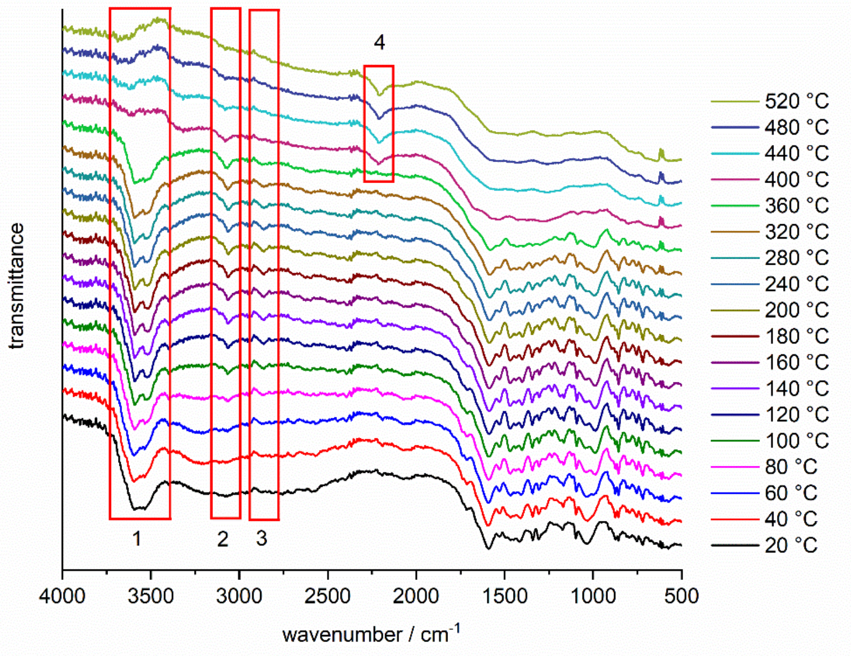

Figure S19: Temperature dependent FT-IR measurement of Al-CAU-10-$L^{0,2,4,6}$ some characteristic bands are marked with numbers from 1 to 4.

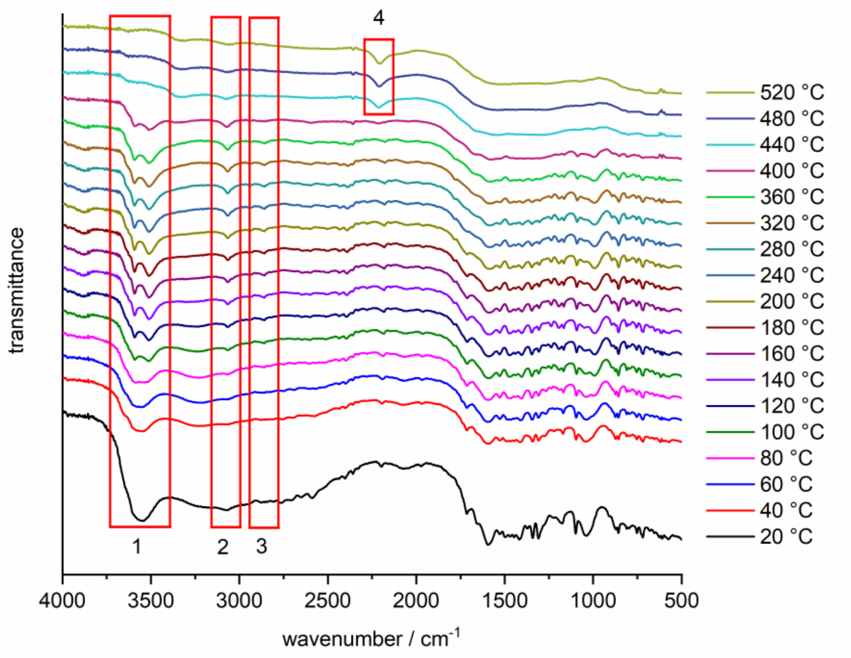

Figure S20: Temperature dependent FT-IR measurement of Ga-MIL-53-L^2_lp1 some characteristic bands are marked with numbers from 1 to 4.

IR-spectra of Al-CAU-10-$L^{0,2,4,6}$, Ga-MIL-53-L^2_lp1 and Ga-MIL-53-L^2_lp2 were also collected on a Bruker ALPHA-FT-IR A220/D-01 using an ATR-unit and they are shown in Fig. S19-S20. Assignments of characteristic vibrational bands are shown in Table S3-5.

All spectra show the presence of nitro groups with characteristic antisymmetric and symmetric stretching vibrations located at 1535 (in solution 1550 cm^{-1}) and 1340 cm^{-1}, respectively.[18] Additionally a very prominent band at 1020 cm^{-1} typical for the C-N stretching vibration of aromatic nitro compounds can be identified. Stretching vibrations of the aromatic ring are observed at 1445 cm^{-1}. The bands at 1580 and 1400 cm^{-1} can be assigned to the antisymmetric and symmetric stretching vibration of the carboxylate group, respectively. Multiple vibrational bands in the range of 3375-3700 cm^{-1} are caused by the -OH stretching vibration of crystal water and the bridging hydroxyl groups in the IBUs. The lower intensity of these bands in the spectra of Ga-MIL-53-L^2_lp2 correlates well with the reduced water content as determined by CHNS analysis when compared to Ga-MIL-53-L^2_lp1.

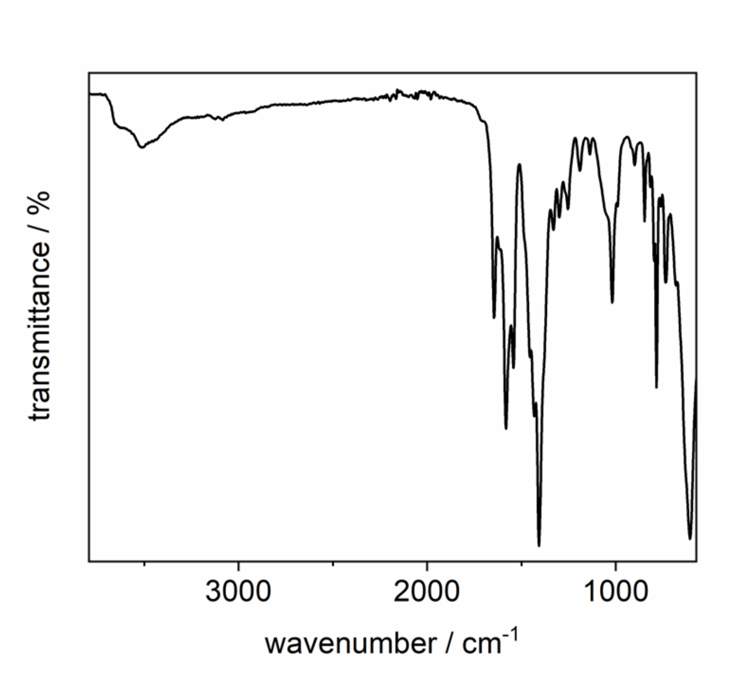

Figure S21: IR-spectra of Al-CAU-10-$L^{0,2,4,6}$.

Table S3: Assignment the vibrational bands in the IR-spectra of Al-CAU-10-$L^{0,2,4,6}$.[19]

Functional group	Intensity	Observed value / cm^{-1}	Vibration
**-C-N**	s	1020	ν(-C-N) symmetric stretch
**$-NO_2$**	m	1340	ν($-NO_2$) symmetric stretch
**-C=O**	vs	1405	ν(-C=O) symmetric stretch
**-C=C-**	w	1445	ν(-C=C-) stretch
**$-NO_2$**	vs	1535	ν($-NO_2$) antisymmetric stretch
**-C=O**	m	1580	ν(-C=O) antisymmetric stretch
**-OH**	vw	3080	ν(-OH) stretch
**-OH**	w	3515	ν(-OH) stretch

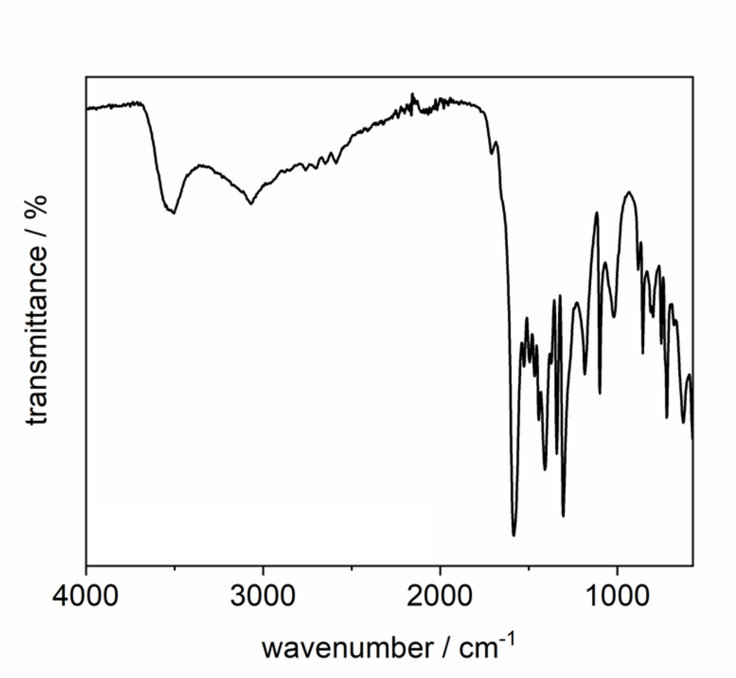

Figure S22: IR-spectra of Ga-MIL-53-L^2_lp1.

Table S4: Assignment the vibrational bands in the IR-spectra of Ga-MIL-53-L^2_lp1.[19]

Functional group	Intensity	Observed value / cm^{-1}	Vibration
**-C-N**	s	1020	ν(-C-N) symmetric stretch
**-NO_2**	m	1336	ν(-NO_2) symmetric stretch
**-C=O**	vs	1405	ν(-C=O) symmetric stretch
**-C=C-**	w	1442	ν(-C=C-) stretch
**-NO_2**	vs	1535	ν(-NO_2) antisymmetric stretch
**-C=O**	m	1580	ν(-C=O) antisymmetric stretch
**-OH**	w	2756, 2700, 2654, 2585	ν(-OH) stretch, intramolecular H-bond
**-OH**	w	3080	ν(-OH) stretch
**-OH**	w	3525	ν(-OH) stretch

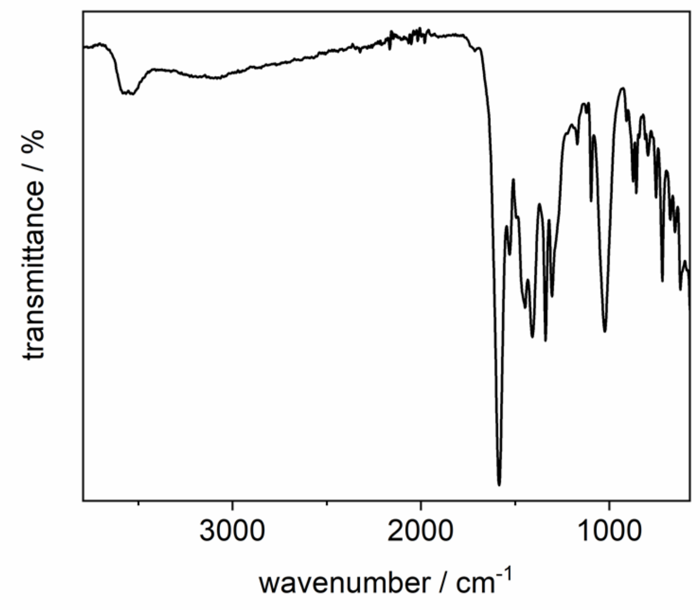

Figure S23: IR-spectra of Ga-MIL-53-L^2_lp2.

Table S5: Assignment the vibrational bands in the IR-spectra of Ga-MIL-53-L^2_lp2.[19]

Functional group	Intensity	Observed value / cm^{-1}	Vibration
**-C-N**	s	1020	ν(-C-N) symmetric stretch
**-NO_2**	m	1336	ν(-NO_2) symmetric stretch
**-C=O**	vs	1405	ν(-C=O) symmetric stretch
**-C=C-**	w	1442	ν(-C=C-) stretch
**-NO_2**	vs	1535	ν(-NO_2) antisymmetric stretch
**-C=O**	m	1580	ν(-C=O) antisymmetric stretch
**-OH**	w	3529	ν(-OH) stretch
**-OH**	w	3589	ν(-OH) stretch

7. Sorption Isotherms

The result of the nitrogen and water vapor sorption measurements are shown in Fig. S24-S27. In addition PXRD patterns of the samples collected before and after the measurements are presented (Fig. S28-S29). The activation temperature and time are listed in Table S6.

Table S6: Activation temperature and time for Al-CAU-10-$L^{0,2,4,6}$ and Ga-MIL-53-L^2_lp1. Activation was carried out under reduced pressure (dynamic vacuum, $p < 10^{-2}$ mbar).

Compound	T / °C	t / h
**Al-CAU-10-$L^{0,2,4,6}$**	180	16 h
**Ga-MIL-53-L^2_lp1**	240	4 h

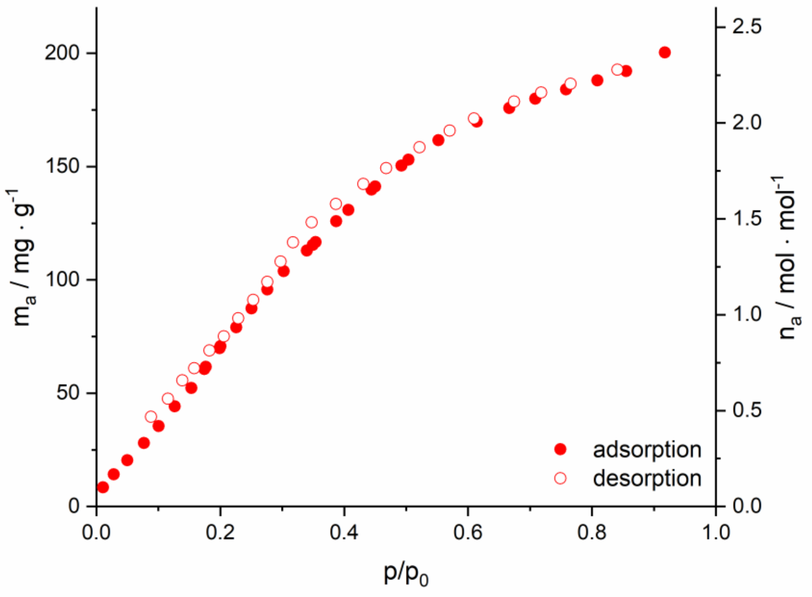

Figure S24: Water vapor sorption isotherm of Al-CAU-10-$L^{0,2,4,6}$ measured at 298 K.

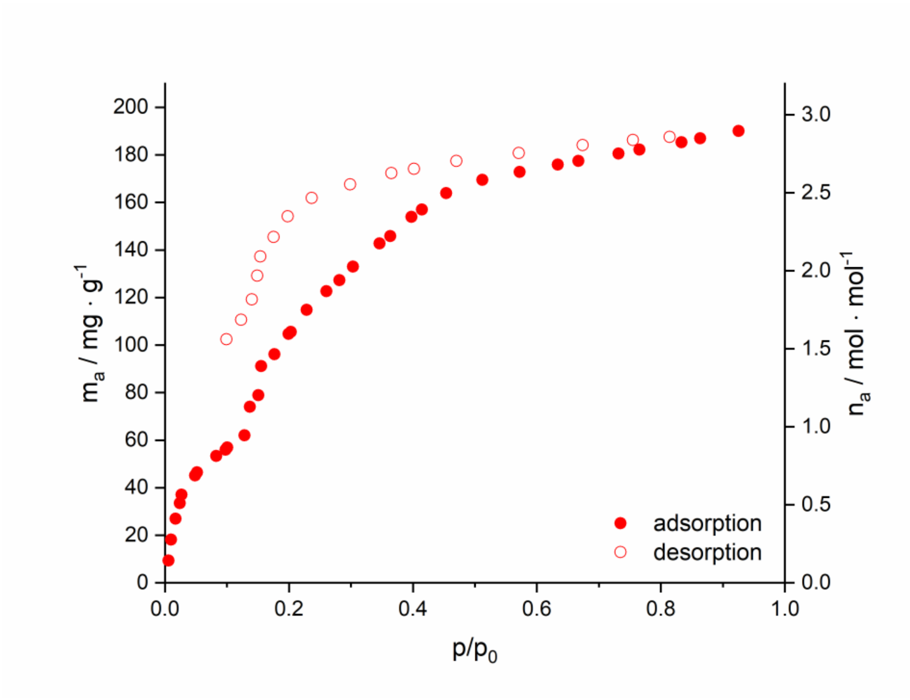

Figure S25: Water vapor sorption isotherm of Ga-MIL-53-L^2_lp1 measured at 298 K.

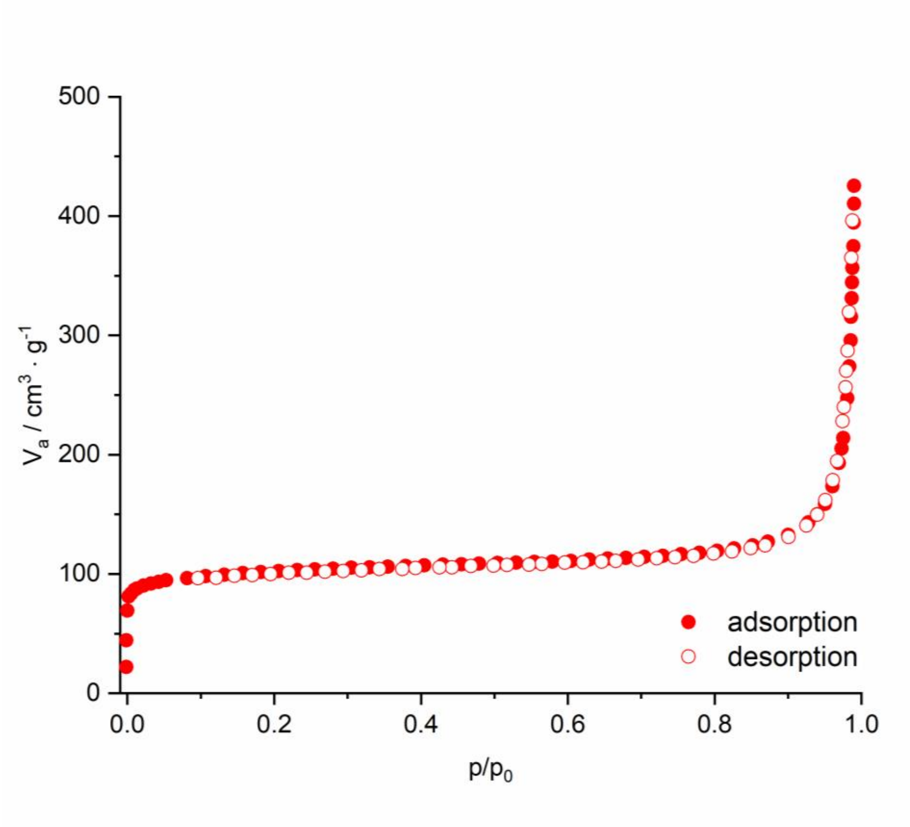

Figure S26: Nitrogen sorption isotherm of Al-CAU-10-L^{0,2,4,6} measured at 77 K.

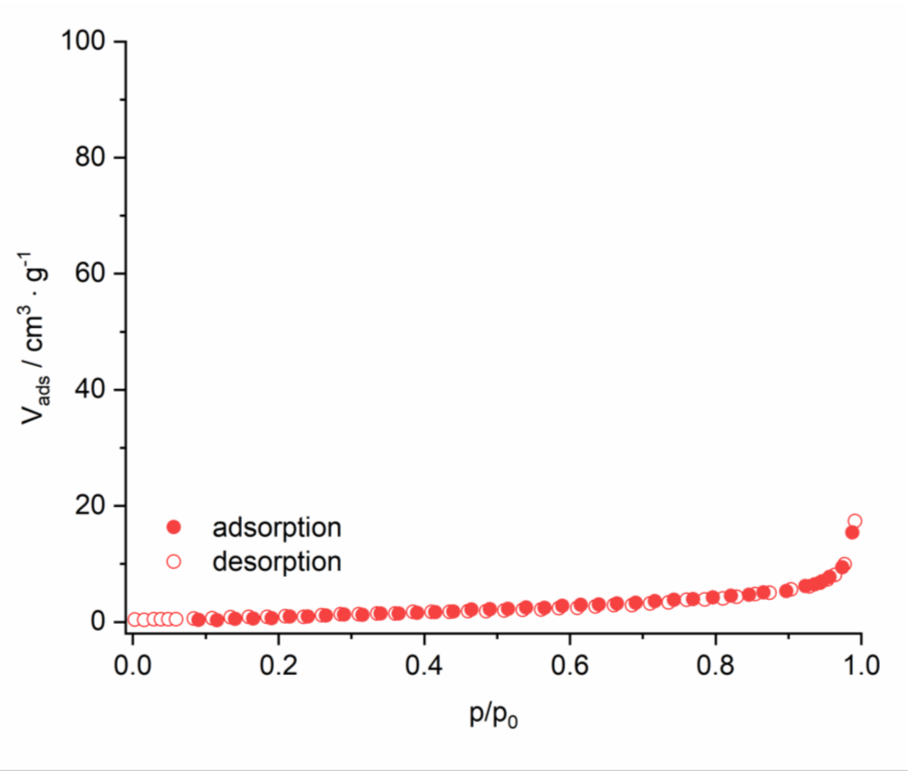

Figure S27: Nitrogen sorption isotherm of Ga-MIL-53-L^2_lp1 measured at 77 K with a BET surface area of a_{SBET} = 3 m^2/g and a micropore volume of V_m = 0.003 cm^3/g.

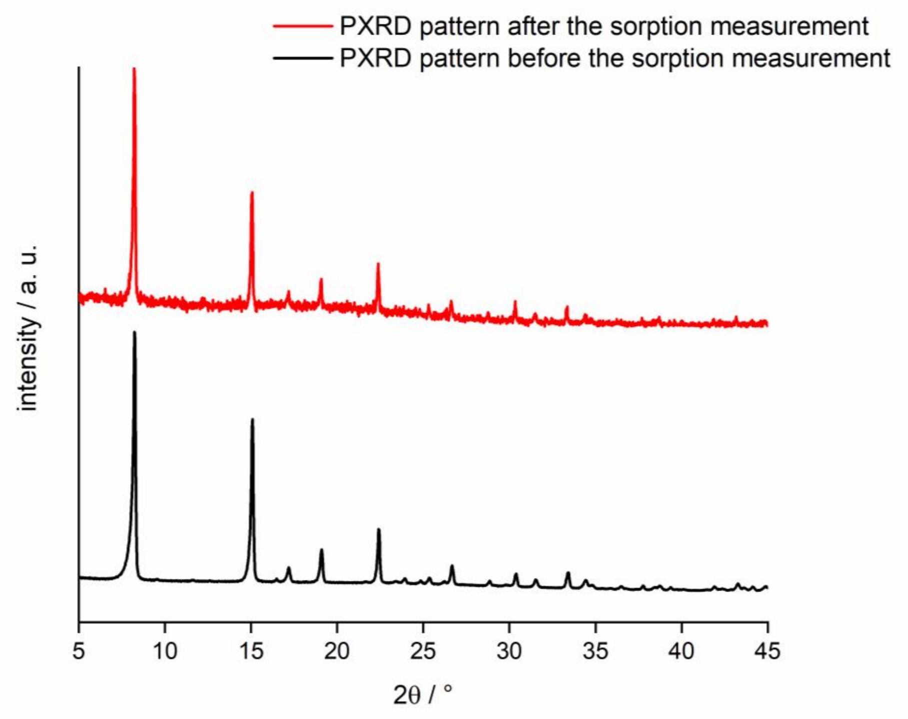

Figure S28: PXRD pattern of Al-CAU-10-$L^{0,2,4,6}$ before and after the sorption measurement. Activation was carried out under reduced pressure (dynamic vacuum <10^{-2} mbar) at 180 °C for 16 h.

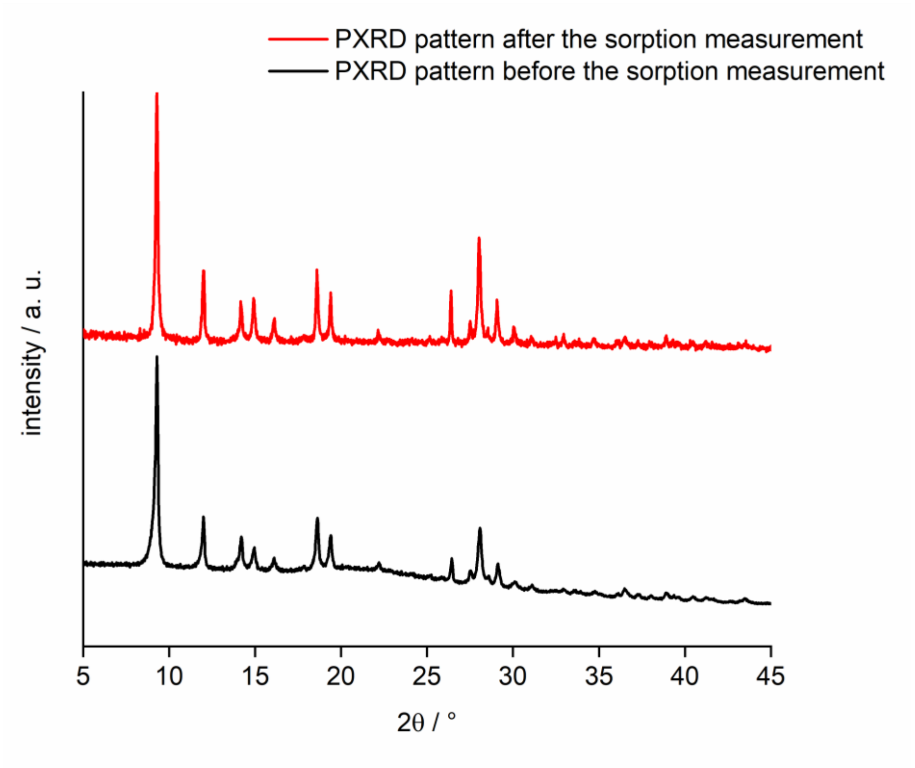

Figure S29: PXRD pattern of Ga-MIL-53-L^2_lp1 before and after the sorption measurement. Activation was carried out under reduced pressure (dynamic vacuum, p <10^{-2} mbar) at 240 °C for 4 h.

8. Thermogravimetric measurement of $Ga(NO_3)_3 \cdot x\ H_2O$

In order to study the water content of $Ga(NO_3)_3 \cdot xH_2O$, thermogravimetric measurements were carried out, resulting in seven water molecules per sum formula (Fig. S30, Tab. S7). The residue identified by PXRD as crystalline *ß*-Ga_2O_3 had formed (Fig. S31). The device used for this measurement was a Linseis STA PT 1000 (nitrogen flow = 8 dm^3/h, heating rate = 4K/min).

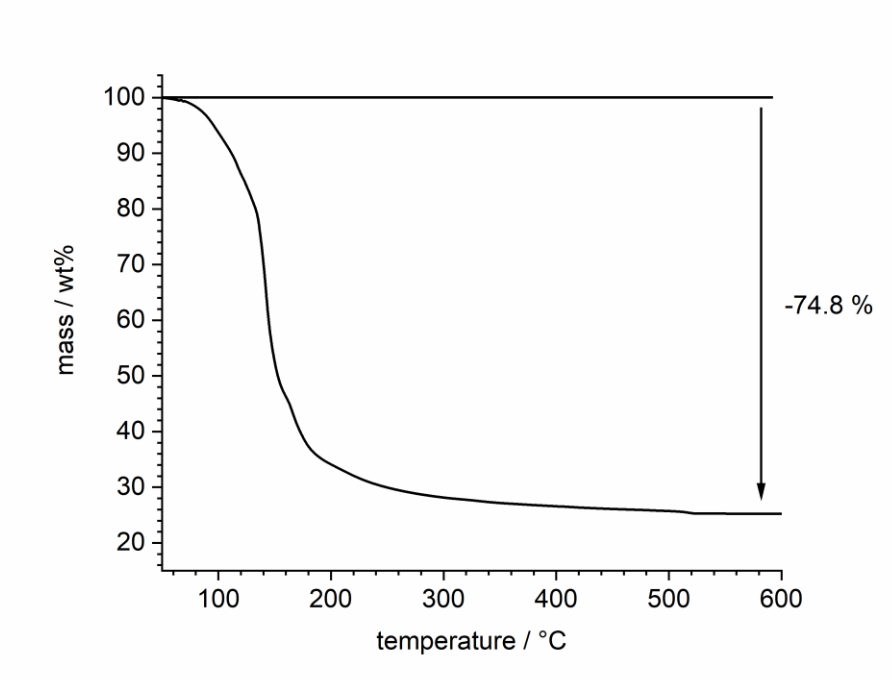

Figure S30: TG curve of $Ga(NO_3)_3 \cdot xH_2O$.

Table S7: Result of the thermogravimetric analysis of $Ga(NO_3)_3 \cdot xH_2O$.

**Product/Step**	**calcd / %**	**obs / %**	**ΔT / °C**
**7 H_2O, 6 NO***	75.5	74.8	25-160
**Ga_2O_3**	24.5	25.2	160-1000

*The measurement was carried out under a nitrogen flow and therefore it is anticipated that the remaining oxygen formed NO molecules.

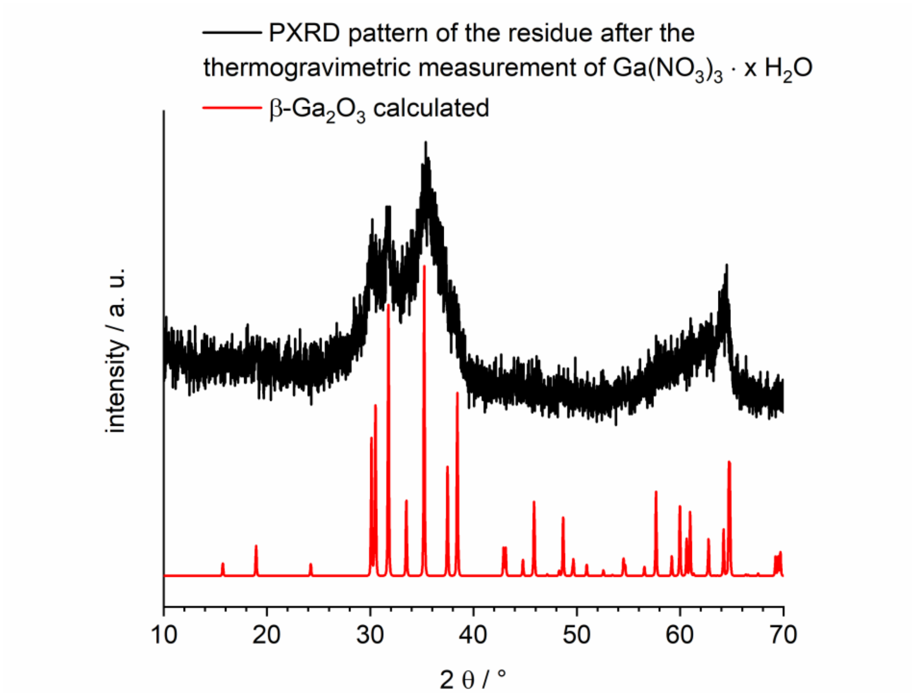

Figure S31: PXRD pattern of the thermal decomposition product of $Ga(NO_3)_3{\cdot}xH_2O$ after the thermogravimetric measurement (black) and a calculated PXRD pattern of ß-Ga_2O_3.[20]

9. GCMC preferential arrangements of the adsorbed water

GCMC preferential arrangements of the adsorbed water molecules within the pores of Ga-MIL-53-L^2_np and _lp1 form are depicted in Figure S32 and Figure S33, respectively.

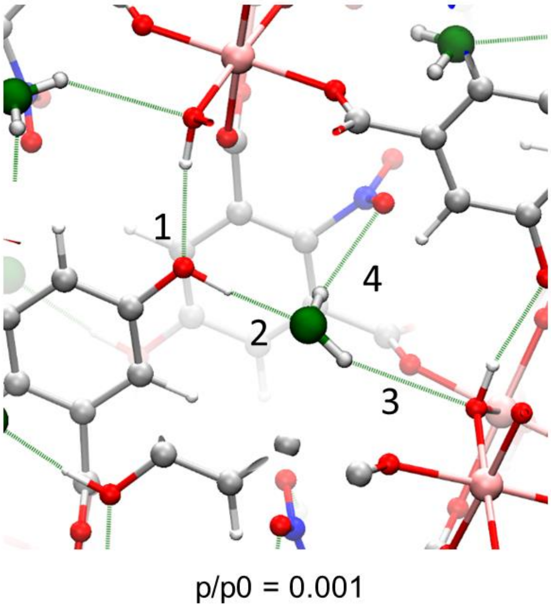

Figure S32: GCMC derived preferential arrangement of the adsorbed water molecules within the poresy of Ga-MIL-53-L^2_np. The very first adsorbed water molecules interact with µ-OH, -OH and -NO_2 sites forming strong hydrogen bonds as labeled as 3, 2, and 4, respectively. 1 refers to the intra-framework O(µ-OH)…O(OH) hydrogen bonds. Atom color code: C – grey, O – red, H – white, Ga – pink. The oxygen atoms of the guest water molecules were plotted in green for easy distinction against framework oxygen atoms.

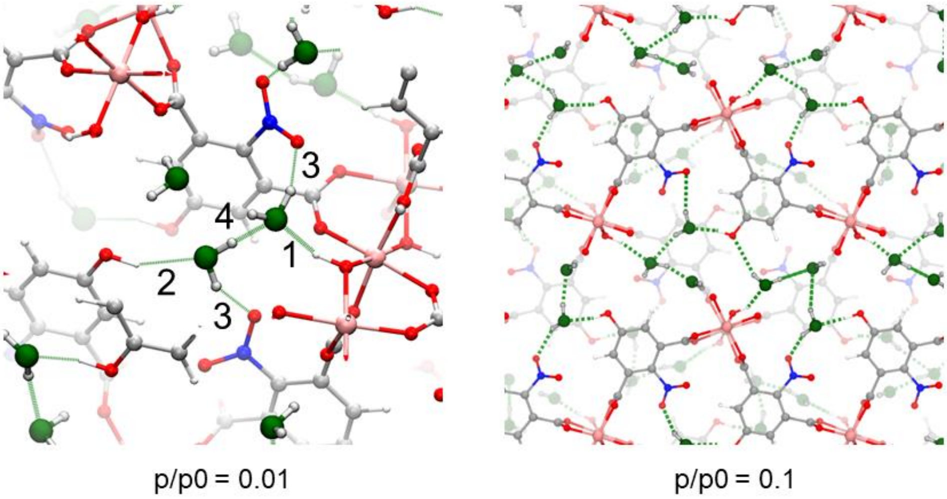

Figure S33: GCMC preferential arrangements of the adsorbed water molecules within the pores of Ga-MIL-53-L^2_lp1. The left panel shows the adsorbed water molecules at $p/p_0 = 0.01$, interacting with adjacent µ-OH, -OH and NO_2 sites as well as other guest water molecules forming hydrogen bonds labeled as 1, 2, 3, and 4, respectively. The right panel shows the typical hydrogen bonded clusters of adsorbed water molecules near the saturation regime at $p/p_0 = 0.1$. Atom color code: C – grey, O –red, H – white, Ga – pink. The oxygens of the guest water molecules were plotted in green for easy distinction against framework oxygens.

10. 3DED data, Rietveld refinements, Le Bail fit, asymmetric units and bond lengths

All indexing and refinement steps of PXRD patterns described herein were carried out using TOPAS[21] Academic. The software used for structural modelling and optimization by force-field calculations was Materials Studio.[22]

Al-CAU-10-L^{0,2,4,6}

For the PXRD data collection, a glass capillary was filled with Al-CAU-10-L^{0,2,4,6}, activated at 220 °C under reduced pressure ($p < 10^{-2}$ mbar) for 4 h and sealed afterwards.

The pattern could be indexed in a tetragonal unit cell with a possible space group symmetry $I4_1/amd$ (No. 141), thus being closely related to CAU-10-CH_3[23] with larger unit cell parameters (a and b = 10.55(7) Å and c = 10.38(3) Å). Thus the crystal structure of CAU-10-CH_3 was used as a starting model. The software Materials Studio was used to add nitro groups to the aromatic ring of the 5-hydroxyphthalate ions in 2-, 4-, and 6 positions. Occupancies were obtained from ^{1}H-NMR spectra and by the integral ratios of the corresponding signals. After imposing the indexed unit cell parameters the structure was subsequently optimized using universal force-field calculations. The structure model was refined with respect to PXRD data by the Rietveld method.[24] Residual electron density in the pore space was identified by Fourier synthesis and attributed to partially occupied oxygen atoms, which serve as placeholders for guest molecules such as water. The linker molecule was treated as a rigid body, hence no standard derivations are given. All other atoms were freely refined using only distance restrains and element specific temperature factors. The final plot is shown in Fig. S34, the asymmetric unit in Fig. S35 and some relevant bond distances in Tab. S8.

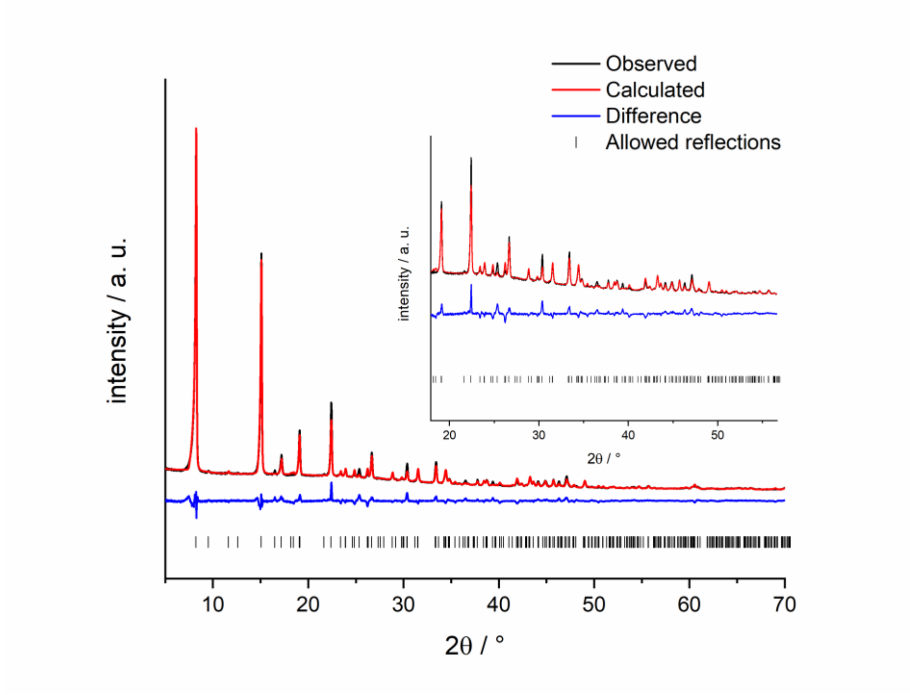

Figure S34: Final Rietveld plot for Al-CAU-10-L^{0,2,4,6}. The observed curve in black, the calculated curve in red, the difference curve in blue and the positions of allowed reflections as black lines.

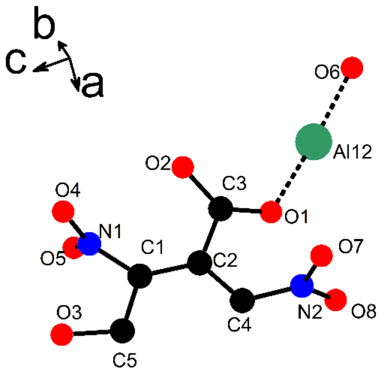

Figure S35: Asymmetric unit of Al-CAU-10-L^{0,2,4,6}.

Table S8: Selected atoms and bond lengths for Al-CAU-10-L^{0,2,4,6}. The slightly different C-C values of the aromatic ring is a result from the force field calculations that were performed to set up the structure model and the fact that this part of the structure was refined as a rigid body.

Atom #1	Atom #2	bond lengths / Å
**Al1**	O1	1.97(1)
	O2	1.89(1)
	O6	1.94(1)
**C1**	N1	1.37
	C2	1.39
	C5	1.41
**C2**	C4	1.41
	C3	1.52(1)
**C4**	N2	1.38
**C5**	O3	1.45
**N1**	O4	1.25
	O5	1.24
**N2**	O7	1.22
	O8	1.26

Three-dimensional electron diffraction (3DED) measurements of Ga-MIL-53-L^2

Three-dimensional electron diffraction data of small single crystals of Ga-MIL-53-L^2 (< 1 µm, Tab. S9 and Fig. S36, respectively) were collected using a JEOL JEM2100 TEM, equipped with a Timepix detector from Amsterdam Scientific Instruments, while continuously rotating the crystal at 0.45° s−1. The experiments were carried out using Instamatic,[25] with data reduction performed by XDS.[26] The acquired intensities were then used to solve the structures with SHELXT,[27] and refined using SHELXL,[28] with electron scattering factors provided by SIR2014.[29]

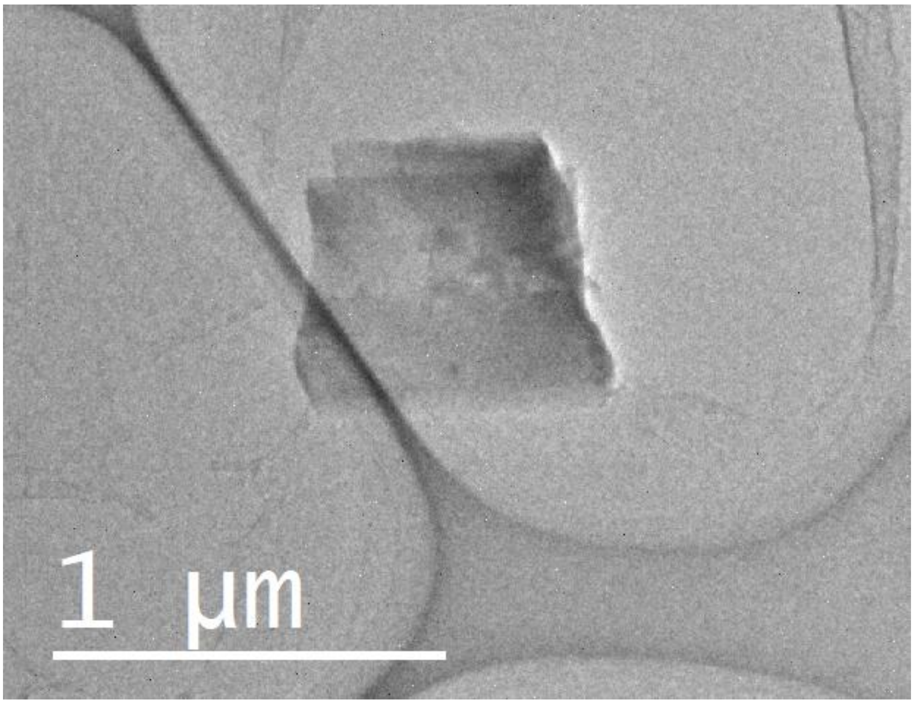

Figure S36: Small single crystal of Ga-MIL-53-L^2_np used for the 3DED measurements.

Table S9: Crystallographic table for 3DED data of Ga-MIL-53-L^2_np.

Parameter	Ga-MIL-53-L^2_np
**Empirical formula**	[C_8GaO_6]
**Wavelength**	0.0251 Å
**Crystal system**	Orthorhombic
**Space group**	*Pnma* (No. 62)
**Unit cell dimensions**	a = 14.84 Å<br>b = 7.09 Å<br>c = 11.64 Å
**Volume**	1223 Å^3
**Z**	2
**Rotation range**	114.58° (-59.64 to 55.12°)
**Index ranges**	$-18 \leq h \leq 18$<br>$-8 \leq k \leq 8$<br>$-13 \leq l \leq 13$
**Reflections collected**	5024
**Independent reflections**	1209<br>[R(int) = 0.1685]
**Completeness (to 0.8 Å resolution)**	88.2 %
**R_1 (ED model) [I > 2σ(I)]**	0.2793

Ga-MIL-53-L^2_lp2

For the PXRD data collection, a glass capillary was filled with Ga-MIL-53-L^2_lp1, activated at 130 °C under reduced pressure (p < 10^{-2} mbar) for 1 h and sealed afterwards.

The pattern could be indexed in an orthogonal unit cell with a possible space group symmetry *Pnma* (No. 62) and very similar cell parameters compared to Ga-MIL-53-L^2_np. Thus the crystal structure of Ga-MIL-53-L^2_np was used as a starting model. The indexed unit cell parameters were imposed and the optimized model was subsequently refined by the Rietveld method. The carbon, nitrogen and oxygen atoms of the linker molecule were treated as rigid body, hence no standard deviation is given. All other atoms were freely refined using only distance restrains and element specific temperature factors. Residual electron density in the pores was identified by Fourier synthesis and attributed to partially occupied oxygen atoms, which serve as placeholder for guest molecules such as water. To account for preferred orientation and peak broadening, a 8th order spherical harmonics function was used. The final plot is shown in Fig. S37 along with the asymmetric unit in Fig. S38 and some relevant bond distances in Tab. S10.

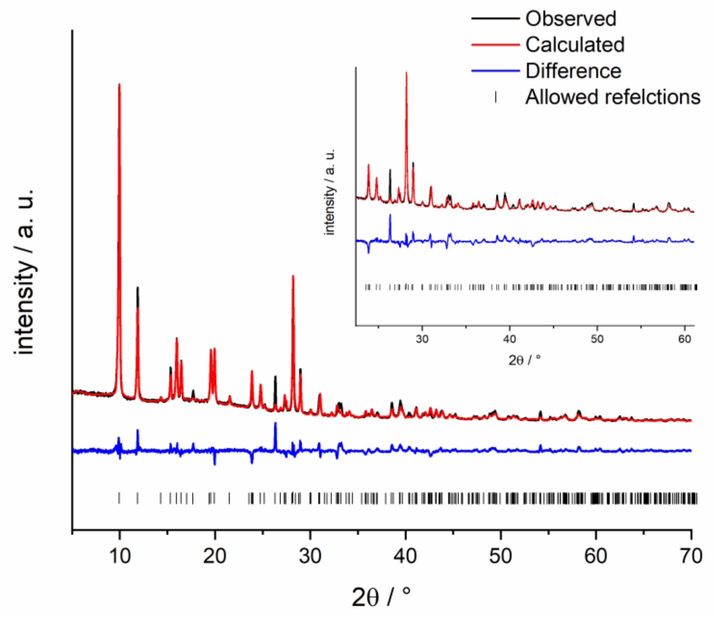

Figure S37: Final plot of the Rietveld refinement for Ga-MIL-53-L^2_lp2. The observed curve in black, the calculated curve in red, the difference curve in blue and the positions of allowed reflections as black lines.

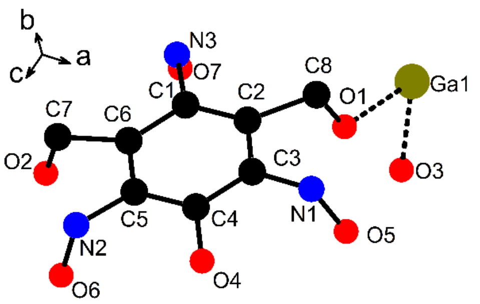

Figure S38: Asymmetric unit of Ga-MIL-53-L^2_lp2.

Table S10: Selected atoms and bond lengths for Ga-MIL-53-L^2_lp2. The slightly different C-C values of the aromatic ring is a result from the force field calculations that were performed to set up the structure model and the fact that this part of the structure was refined as a rigid body.

Atom #1	Atom #2	bond lengths / Å
**Ga1**	O1	1.99(2)
	O2	1.95(2)
	O3	1.96(2)
**C1**	N3	1.39
	C2	1.41
	C6	1.40
**C2**	C8	1.50(5)
	C3	1.39
**C3**	N1	1.38
	C4	1.40
**C4**	O4	1.47
**C4**	C5	1.40
**C5**	N2	1.39
	C6	1.40
**C6**	C7	1.49(6)
**C7**	O2	1.26(3)
**C8**	O1	1.27(3)
**N1**	O5	1.23
**N2**	O6	1.24
**N3**	O7	1.24

Ga-MIL-53-L^2_np

For the PXRD data collection, a glass capillary was filled with Ga-MIL-53-L^2_lp1, activated at 240 °C under reduced pressure ($p < 10^{-2}$ mbar) for 4 h and sealed afterwards.

The crystal structure model as determined from electron diffraction data was modified in Materials Studio by adding nitro groups with the occupancy factors calculated from the ^{1}H-NMR spectra to the aromatic ring. After that, the structure model was optimized by force-field calculations and subsequently refined by the Rietveld method. The carbon, nitrogen and oxygen atoms of the linker molecule were treated as rigid body, hence no standard deviation is given. All other atoms were freely refined using only distance restrains and element specific temperature factors. The final plot is shown in Fig. S39 along with the asymmetric unit in Fig. S40 and some relevant bond distances in Tab. S11.

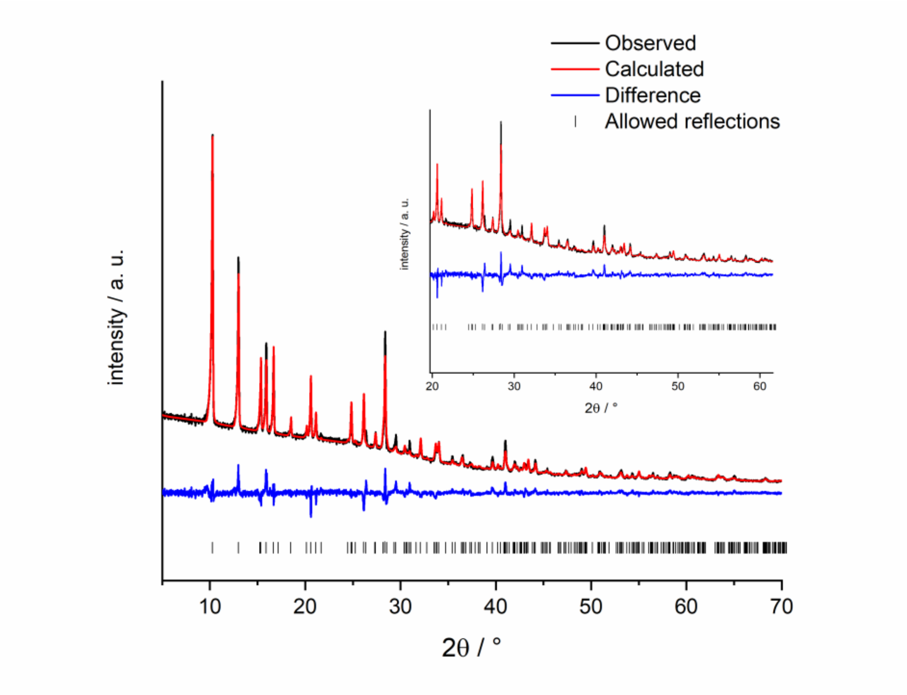

Figure S39: Final plot of the Rietveld refinement for Ga-MIL-53-L^2_np. The observed curve in black, the calculated curve in red, the difference curve in blue and the positions of allowed reflections as black lines.

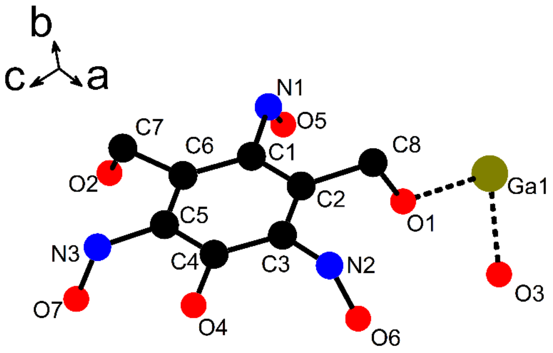

Figure S40: Asymmetric unit of Ga-MIL-53-L^2_np.

Table S11: Selected atoms and bond lengths for Ga-MIL-53-L^2_np. The slightly different C-C values of the aromatic ring is a result from the force field calculations that were performed to set up the structure model and the fact that this part of the structure was refined as a rigid body.

Atom #1	Atom #2	bond lengths / Å
**Ga1**	O1	2.00(1)
	O2	1.98(1)
	O3	2.02(1)
**C1**	C2	1.39
	C6	1.39
	N1	1.40
**C2**	C3	1.41
	C8	1.48(3)
**C3**	C4	1.39
	N2	1.36
**C4**	C5	1.39
	O4	1.46
**C5**	N3	1.39
	C6	1.40
**C6**	C7	1.52(4)
**C7**	O2	1.27(2)
**C8**	O1	1.28(2)
**N1**	O5	1.22
**N2**	O6	1.22
**N3**	O7	1.25

Ga-MIL-53-L^2_lp1

The pattern could be indexed in a orthogonal unit cell with a possible space group symmetry *Pnma* (No. 62), thus being symmetrically related to [Sc(OH)(BDC)][30] (with H_2BDC = benzene-1,4-dicarboxylic acid). Thus the crystal structure of [Sc(OH)(BDC)] was used as a starting model. The software Materials Studio was used to substitute the $BPDC^{2-}$ ions to 5-hydroxyphthalate ions with nitro groups in 2-, 4-, and 6 positions. Occupancies were calculated from ^{1}H-NMR spectra (section 2) and the integral ratios of the corresponding signals. After imposing the indexed unit cell parameters the structure was subsequently optimized using universal force-field calculations. The structure could not be refined with respect to PXRD data, most probably because of disordered residual electron density in the pores. A Le Bail-fit[31], Fig. S41, was carried out to confirms the phase purity and possible space group $Pna2_1$. The comparison between the calculated and measured PXRD pattern, the structure models asymmetric unit and some relevant bond lengths are shown in Figure S42, S43 and Table S12, respectively.

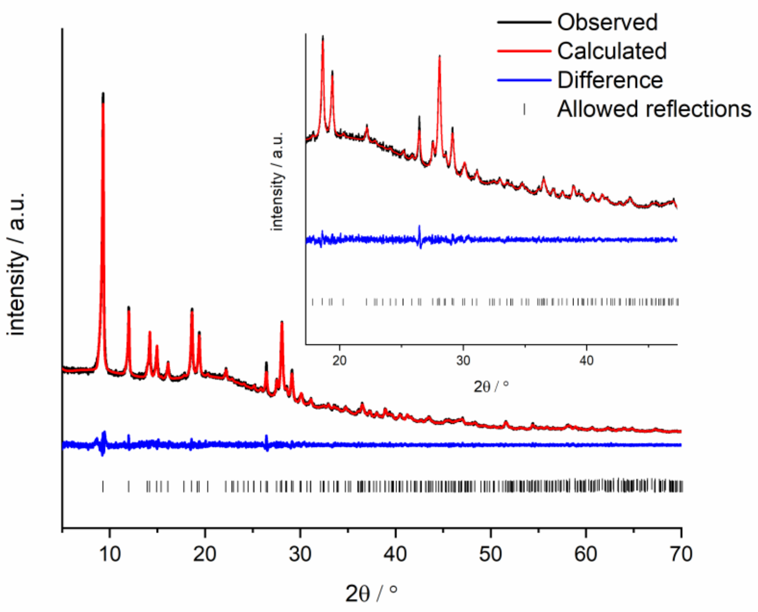

Figure S41: Le Bail plot for Ga-MIL-53-L^2_lp1. The observed curve in black, the calculated curve in red, the difference curve in blue and the positions of allowed reflections as black lines.

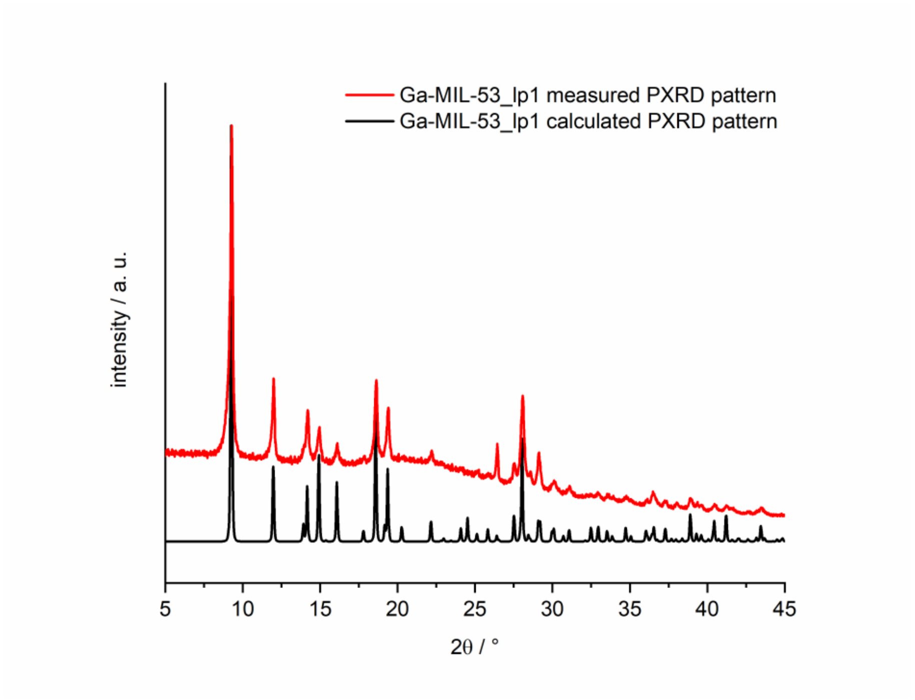

Figure S42: Comparison between the calculated and the experimental PXRD pattern of Ga-MIL-53-L^2_lp1.

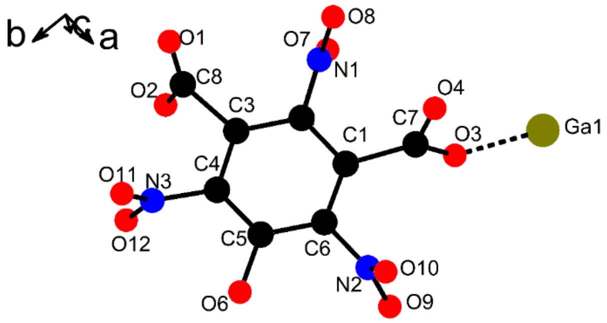

Figure S43: Asymmetric unit of Ga-MIL-53-L^2_lp1.

Table S12: Selected atoms and bond lengths for Ga-MIL-53-L^2_lp1.

Atom #1	Atom #2	bond lengths / Å
**Ga1**	O1	2.00
	O2	2.00
	O3	2.01
	O4	2.04
	O5	1.97
**C1**	C7	1.54
**C2**	C3	1.43
	C1	1.42
**C3**	C4	1.40
	C8	1.56
**C4**	C5	1.40
	N3	1.40
**C5**	C6	1.39
	O6	1.39
**C6**	N2	1.41
	C1	1.39
**N1**	O7	1.29
	O8	1.26
**N2**	O9	1.26
	O10	1.27
**N3**	O11	1.24
	O12	1.28

Synthesis and Rietveld refinement of Ga-MIL-53-BDC-NO_2

For the synthesis of Ga-MIL-53-BDC-NO_2 20.0 mg H_2BDC-NO_2, 900 µL deionized water, 50 µL NaOH (2 mol/L) and 50 µL of an aqueous solution of gallium nitrate heptahydrate (0.72 mol/L) were mixed in a 6 mL Pyrex® glass vial under stirring for 30 seconds at maximum rate. The suspension was heated in an aluminum block for 1 h at 120 °C and after cooling to room temperature the white product was separated by centrifugation in a 3 ml vial at 9000 rpm for 3 min. Remaining residues of H_2BDC-NO_2 were removed by washing two times with methanol (redispersion and centrifugation). The white solid was dried at 80 °C for 1 h. For characterization by PXRD the dry product was filled in a capillary, which was sealed, after activating the product at 220 °C for 1 h under reduced pressure ($p < 10^{-2}$ mbar).

The pattern could be indexed in an orthogonal unit cell with a possible space group symmetry *Pnma* (No. 62) and similar cell parameters compared to Ga-MIL-53-L^2_np. Hence, the structure model of Ga-MIL-53-L^2_np was used as a starting model, which was modified in Materials Studios by removing all functional groups beside the nitro group in the 2-position. The indexed unit cell parameters were imposed and the optimized model was subsequently refined by the Rietveld method. The carbon, nitrogen and oxygen atoms of the linker molecule were treated as rigid body, hence no standard deviation is given. All other atoms were freely refined using only distance restrains and element specific temperature factors. Residual electron density in the pores was identified by Fourier synthesis and attributed to partially occupied oxygen atoms, which serve as placeholder for guest molecules such as water. The final plot is shown in Fig. S44 along with the asymmetric unit in Fig. S45, some relevant bond distances in Tab. S13 and its crystal structure in Fig. S46.

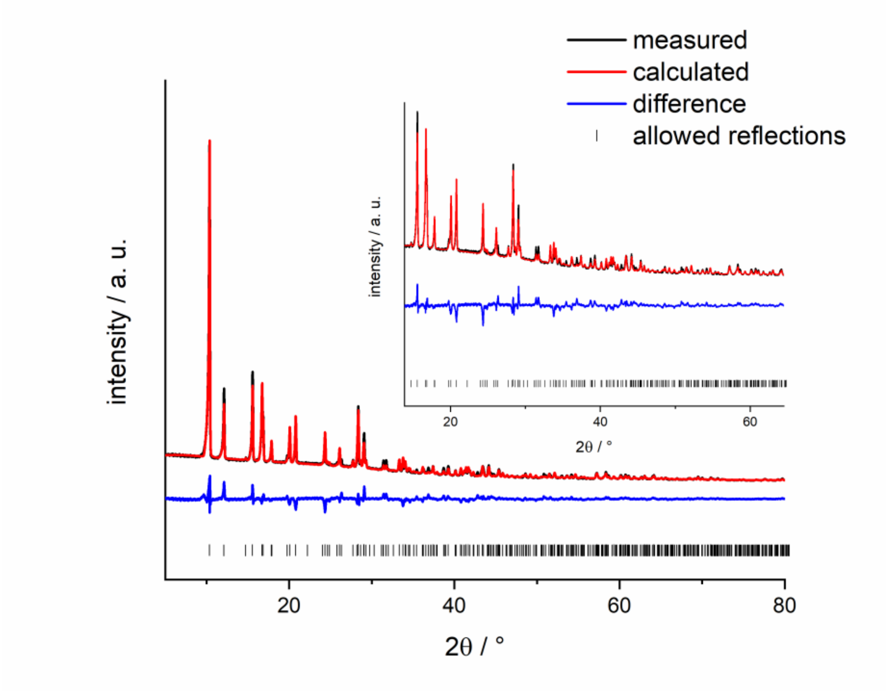

Figure S44: Final plot of the Rietveld refinement for Ga-MIL-53-BDC-NO_2. The observed curve in black, the calculated curve in red, the difference curve in blue and the positions of allowed reflections as black lines.

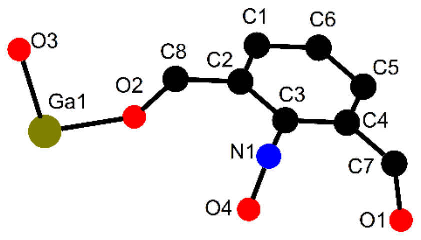

Figure S45: Asymmetric unit of Ga-MIL-53-BDC-NO_2.

Table S13: Selected atoms and bond lengths for Ga-MIL-53-BDC-NO_2.

Atom #1	Atom #2	bond lengths / Å
**Ga1**	O1	1.95(1)
	O2	1.95(1)
	O3	2.02(1)
**C1**	C2	1.40
	C6	1.40
**C2**	C3	1.40
	C8	1.48(3)
**C3**	C4	1.40
	N1	1.39
**C4**	C5	1.40
	C7	1.48(1)
**C5**	C6	1.40
**C7**	O1	1.27(1)
**C8**	O2	1.27(1)
**N1**	O4	1.22

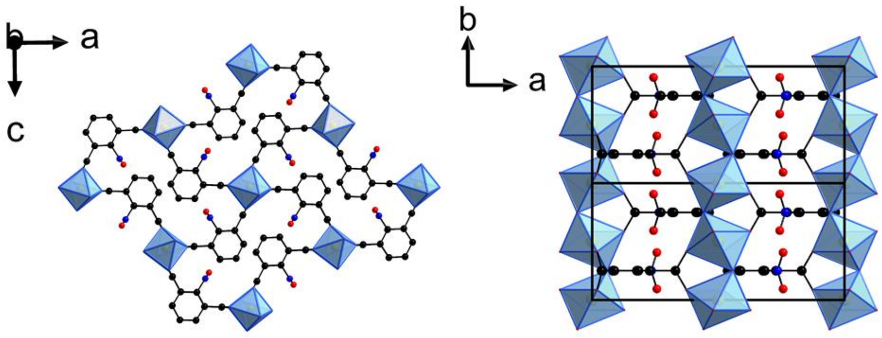

Figure S46: Crystal structure of Ga-MIL-53-BDC-NO2 along [010] (left) and a 2x1 super cell along [001] (right).

11. References

(1) J. VandeVondele, M. Krack, F. Mohamed, M. Parrinello, T. Chassaing, J. Hutter, *Computer Physics Communications* **2005**, *167*, 103–128.

(2) J. Hutter, M. Iannuzzi, F. Schiffmann, J. VandeVondele, Wiley *Interdisciplinary Reviews: Computational Molecular Science* **2014**, *4*, 15–25.

(3) The CP2K developers group http://www.cp2k.org (accessed Feb 10, 2019).

(4) J. P. Perdew, K. Burke, M. Ernzerhof, *Physical Review Letters* **1996**, *77*, 3865–3868.

(5) S. Grimme, J. Antony, S. Ehrlich, H. Krieg, *Journal of Chemical Physics* **2010**, *132*, 154104.

(6) S. Grimme, *Journal of Computational Chemistry* **2004**, *25*, 1463–1473.

(7) J. VandeVondele, J. Hutter, *Journal of Chemical Physics* **2007**, *127*, 114105.

(8) S.Goedecker, M. Teter, J. Hutter, *Physical Review B* **1996**, *54*, 1703–1710.

(9) M. Krack, *Theoretical Chemistry Accounts* **2005**, *114*, 145–152.

(10) C. Hartwigsen, S. Goedecker, J. Hutter, *Physical Review B* **1998**, *58*, 3641–3662.

(11) D. Dubbeldam, S. Calero, D. E. Ellis, R. Q. Snurr, *Molecular Simulations* **2016**, *42*, 81–101.

(12) H. W. Horn, W. C. Swope, J. W. Pitera, J. D. Madura, T. J. Dick, G. L. Hura, T. Head-Gordon, *Journal of Chemical Physics* **2004**, *120*, 9665–9678.

(13) A. K. Rappe, C. J. Casewit, K. S. Colwell, W. A. Goddard, W. M. Skiff, *Journal of the American Chemical Society* **1992**, *114*, 10024–10035.

(14) W. L. Jorgensen, D. S. Maxwell, J. Tirado-Rives, *Journal of the American Chemical Society* **1996**, *118*, 11225–11236.

(15) C. D. Wick, J. M. Stubbs, N. Rai, J. I. Siepmann, *Journal of Physical Chemistry B* **2005**, *109*, 18974–18982.

(16) A. Cadiau, J. S. Lee, D. Damasceno Borges, P. Fabry, T. Devic, M. T. Wharmby, C. Martineau, D. Foucher, F. Taulelle, C.-H. Jun, et al. *Advanced Materials* **2015**, *27*, 4775–4780.

(17) T. J. H. Vlugt, E. García-Pérez, D. Dubbeldam, S. Ban, S. Calero, *Journal of Chemical Theory and Computation* **2008**, *4*, 1107–1118.

(18) J. Weidlein, U. Müller, K. Dehnicke, Schwingungsspektroskopie: Eine Einführung; Thieme, **1988**.

(19) G. Socrates, *Infrared and Raman Characteristic Group Frequencies: Tables and Charts*; Wiley, **2004**.

(20) D. Dohy, J. R. Gavarri, *Journal of Solid State Chemistry* **1983**, *49*, 107–117.

(21) A. Coelho, *Journal of Applied Crystallography* **2018**, *51*, 210–218.

(22) Accelrys Incorporated. *Materials Studios*; San Diego, **2009**.

(23) H. Reinsch, M. A. van der Veen, B. Gil, B. Marszalek, T. Verbiest, D. E. de Vos, N.Stock, *Chemistry of Materials* **2013**, *25*, 17–26.

(24) H. Rietveld, *Acta Crystallographica* **1967**, *22*, 151–152.

(25) M. O. Cichocka, J. Ångström, B. Wang, X. Zou, S. Smeets, *Journal of Applied Crystallography* **2018**, *51*, 1652–1661.

(26) W. XDS. Kabsch, *Acta Crystallographica Section D Biological Crystallography* **2010**, *66*, 125–132.

(27) G. M. Sheldrick, *Acta Crystallographica Section A Foundations of Crystallography* **2015**, *71*, 3–8.

(28) G. M. Sheldrick, Acta Crystallogr. *Acta Crystallographica Section A Foundations of Crystallography* **2008**, *64*, 112–122.

(29) M. C. Burla, R. Caliandro, B. Carrozzini, G. L. Cascarano, C. Cuocci, C. Giacovazzo, M. Mallamo, A. Mazzone, G. Polidori, *Journal of Applied Crystallography* **2015**, *48*, 306–309.

(30) J. P. S. Mowat, S. R. Miller, A. M.Z. Slawin, V. R. Seymour, S. E. Ashbrook, P. A. Wright, *Microporous and Mesoporous Materials* **2011**, *142*, 322–333.

(31) A. Le Bail, *Powder Diffraction* **2005**, *20*, 316–326.
